# Correcting Basis Set Incompleteness in Wave Function Correlation Energy by Dressing Electronic Hamiltonian with an Effective Short-Range Interaction

**DOI:** 10.1021/acs.jpclett.5c01070

**Published:** 2025-06-17

**Authors:** Michał Hapka, Aleksandra Tucholska, Marcin Modrzejewski, Pavlo Golub, Libor Veis, Katarzyna Pernal

**Affiliations:** † Faculty of Chemistry, University of Warsaw, ul. L. Pasteura 1, 02-093 Warsaw, Poland; ‡ Institute of Physics, 49584 Lodz University of Technology, ul. Wolczanska 217/221, 93-005 Lodz, Poland; ¶ J. Heyrovský Institute of Physical Chemistry, Academy of Sciences of the Czech Republic, v.v.i., Dolejškova 3, 18223 Prague 8, Czech Republic

## Abstract

We propose a general approach to reducing basis set incompleteness error in electron correlation energy calculations. The correction is computed alongside the correlation energy in a single calculation by modifying the electron interaction operator with an effective short-range electron–electron interaction. Our approach is based on a local mapping between the Coulomb operator projected onto a finite basis and a long-range interaction represented by the error function with a local range-separated parameter, originally introduced by Giner et al. [ J. Chem. Phys. 2018, 149, 194301 30466264 10.1063/1.5052714]. Unlike the basis set incompleteness error correction proposed in that work, our method does not rely on short-range correlation density functionals. As a numerical demonstration, we apply the method with complete active space wave functions. Correlation energies are computed using two distinct approaches: the linearized adiabatic connection (AC0) method and *n*-electron valence state second-order perturbation theory (NEVPT2). We obtain encouraging results for the relative energies of test molecules, with accuracy in a triple-ζ basis set comparable to or exceeding that of uncorrected AC0 or NEVPT2 energies in a quintuple-ζ basis set.

## Graphical abstract

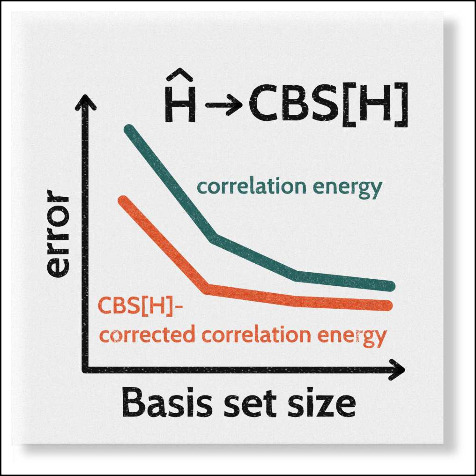

Many-electron wave function theories (WFT) provide a powerful framework for predicting the physical and chemical properties of matter. However, their accuracy is inherently limited by the size of the one-electron basis set. The steep computational cost of expanding the basis prevents reaching the so-called complete-basis-set (CBS) limit, which is essential for achieving quantitative agreement with experimental data. At the core of this problem is the slow convergence of the electron correlation energy with the basis set enlargement, which arises primarily from the difficulty in describing short-range correlation effects and the electron–electron cusp.

To address this issue, explicitly correlated (R12, F12) methods have been developed both for single-reference wave function approaches^1–3^ and multireference methods.^4,5^ In the multireference context, these techniques have been incorporated into complete active space (CAS) multireference frameworks and their second-order perturbation corrections, leading to the development of CASPT2-F12 ^6^ and NEVPT2-F12 ^7^ methodologies. Explicitly correlated methods significantly enhance the basis set convergence of the correlation energy, but their implementation comes with notable challenges. The need for an auxiliary basis set to evaluate three-electron integrals introduces additional computational overhead, and the underlying complex formalism requires substantial theoretical developments to integrate these methods into new computational frameworks. A related approach, aiming at improving the basis set convergence of the energy by introducing a correlation factor, is based on transcorrelated (TC) Hamiltonian methodology.^8^ It relies on a similarity transformation of the Hamiltonian, leading to a non-Hermitian operator. TC yields exact energy regardless of the form of the correlation factor only in the complete basis set limit. Various strategies to build effective correlation factors have been proposed for finite basis sets.^9–12^

An alternative approach to addressing the basis set incompleteness (BSI) error was proposed by Giner, Toulouse, and co-workers.^13^ Their method, known as the density-based basis-set correction (DBBSC), is derived by matching the electron–electron Coulomb interaction projected onto a given basis set with a long-range electron interaction. The complementary short-range interaction defines the short-range correlation energy, which is equated to the BSI error. This error is then accounted for using approximate short-range correlation density functionals.^14,15^ DBBSC has been applied to both single-reference 16,17 and multiconfigurational wave function methods.^18,19^ Although it does not outperform explicitly correlated methods, it offers reduced computational cost and memory requirements.^20,21^

In this work, we introduce a novel approach to mitigate the BSI error in the electron correlation energy by modifying the Coulomb Hamiltonian. Specifically, we incorporate an effective, basis set-dependent short-range interaction designed to recover the missing short-range correlation effects arising from basis set limitations. Similar to DBBSC, the method employs an approximate mapping between the full Coulomb potential and a long-range interaction; however, unlike DBBSC, it does not rely on density functional approximations. We demonstrate the efficacy of the method by accelerating the convergence of correlation energy computations using multireference wave function methods, showcasing its potential to improve electronic structure predictions for complex molecular systems.

## Long-Range Electron Interacting Hamiltonian and a Coulomb Hamiltonian in a Finite Basis Set

Let B indicate a finite (given) basis set of one-electron functions. Assume that the orbitals contained in B are orthonormal. The electronic Hamiltonian projected onto the Hilbert space spanned by basis set functions in B , in the second quantization is written as

1 $${Ĥ}_{B}=\overset{B}{\underset{pq}{\sum }}h_{pq}{â}_{p}^{†}{â}_{q}+\frac{1}{2}\overset{B}{\underset{pqrs}{\sum }}g_{pqrs}{â}_{p}^{†}{â}_{q}^{†}{â}_{s}{â}_{r}$$

where indices *pqrs* correspond to spinorbitals. The upper limits of the summations indicate that spatial components of the latter, orbitals {φ_*p*_(**r**)}, belong to B . Therefore, a result of acting with the operators included in the summation on states orthogonal to a space of states spanned by B is null. {*h*_*pq*_} denotes a set of one-electron integrals involving kinetic energy (*t*) and nuclear-electron (υ_ne_) interaction, *h*_*pq*_ = *t*_*pq*_ + [υ_ne_]_*pq*_, and {*g*_*pqrs*_} are two-electron integrals for the Coulomb electron interaction, namely

2 $$g_{pqrs}=\left\langle pq\left|\upsilon_{ee}\right|rs\right\rangle =\int \int \varphi_{p}\left(r_{1}\right)\varphi_{q}\left(r_{2}\right)r_{12}^{-1}\varphi_{r}\left(r_{1}\right)\varphi_{s}\left(r_{2}\right)dr_{1}dr_{2}$$

The ground state energy corresponding to a finite-basis set wave function $\Psi_{B}$ obtained for the Hamiltonian ${Ĥ}_{B}$ differs from the nonrelativistic energy ⟨Ψ|*Ĥ*|Ψ⟩, where *Ĥ* is the exact electronic Hamiltonian,

3 $$Ĥ=\mathrm{T̂}+{\mathrm{V̂}}_{\mathrm{ne}}+{\mathrm{V̂}}_{ee}$$

and Ψ is the exact wave function. This difference defines the BSI correction,

4 $$\varepsilon_{B}^{\mathrm{CBS}}=\left\langle \Psi \left|Ĥ\right|\Psi \right\rangle -\left\langle \Psi_{B}\left|{Ĥ}_{B}\right|\Psi_{B}\right\rangle$$

The correction is negative and its magnitude is related to a deficient description of the electron correlation at short interelectron distances. Thus, $\varepsilon_{B}^{\mathrm{CBS}}$ can be regarded as a short-range correlation energy.

Consider a model Hamiltonian, indicated as *Ĥ* ^LR^, which is free of singularity at electron coalescence, i.e. the Coulomb interaction, υ_*ee*_(*r*_12_)=*r*_12_^–1^, is replaced by the long-range (LR) interaction operator υ_*ee*_^LR^(*r*_12_). By definition, it is finite at *r*_12_ = 0 and decays as *r*_12_^–1^ with interelectron distance

5 $$\underset{r_{12}\to 0}{\lim}r_{12}\upsilon_{ee}^{\mathrm{LR}}\left(r_{12}\right)=0$$

6 $$\underset{r_{12}\to \infty }{\lim}r_{12}\upsilon_{ee}^{\mathrm{LR}}\left(r_{12}\right)=1$$

The real-space representation and the second-quantized form of *Ĥ* ^LR^,

7 $${Ĥ}^{\mathrm{LR}}=\overset{\mathrm{CBS}}{\underset{pq}{\sum }}{\mathrm{h̃}}_{pq}{â}_{p}^{†}{â}_{q}+\frac{1}{2}\overset{\mathrm{CBS}}{\underset{pqrs}{\sum }}g_{pqrs}^{\mathrm{LR}}{â}_{p}^{†}{â}_{q}^{†}{â}_{s}{â}_{r}$$

are equivalent if a complete basis set of one-electron functions is assumed, which is indicated in the upper limits of summations in eq 7. Integrals {*g*_*pqrs*_^LR^} involve the long-range electron interaction, namely *g*_*pqrs*_^LR^ = ⟨*pq*|υ_*ee*_^LR^|*rs*⟩.

By definition, it is assumed that the one-electron part of the LR Hamiltonian, *h̃*, includes a kinetic energy operator and a local potential that fixes the ground state electron density ρ_Ψ^LR^_(**r**) = ⟨Ψ^LR^|ρ̂ (**r**)|Ψ^LR^⟩ to be equal to a given density ρ­(**r**). This potential is a central concept in multiconfigurational DFT.^22–26^ In one of such formulations,^14,15,27^ the energy is obtained as a sum of the expectation value of the exact Hamiltonian with Ψ^LR^ [ρ] which is a ground state of *Ĥ* ^LR^, and the multideterminantal short-range (SR) correlation functional defined as^13^

8 $$E_{c,\mathrm{md}}^{\mathrm{SR}}\left[\rho \right]=\underset{\Psi \to \rho }{\min}\left\langle \Psi \right|\mathrm{T̂}+{\mathrm{V̂}}_{ee}\left|\Psi \right\rangle -\left\langle \Psi^{\mathrm{LR}}\left[\rho \right]\left|\mathrm{T̂}+{\mathrm{V̂}}_{ee}\right|\Psi^{\mathrm{LR}}\left[\rho \right]\right\rangle$$

This functional captures short-range correlation effects missing in Ψ^LR^.

Consider the wave functions Ψ^LR^ and $\Psi_{B}$ corresponding to the long-range Hamiltonian and the exact Hamiltonian projected onto a finite basis, eqs 7 and 1, respectively. Neither of these wave functions exhibits the electron coalescence cusp. Also, recall that by definition the operator υ_*ee*_^LR^(*r*_12_) shares the long-range behavior of the Coulomb interaction, see eq 6. It is therefore reasonable to assume that there exists a basis-set specific long-range interaction operator $\upsilon_{\mathrm{ee}}^{\mathrm{LR},B}$ , such that the wave functions Ψ^LR^ and $\Psi_{B}$ yield the same energies for the exact Hamiltonian in eq 3

$$\exists \upsilon_{ee}^{\mathrm{LR},B}\;\left\langle \Psi^{\mathrm{LR}}\left[\rho^{B}\right]\left|\mathrm{T̂}+{\mathrm{V̂}}_{ee}\right|\Psi^{\mathrm{LR}}\left[\rho^{B}\right]\right\rangle =\left\langle \Psi_{B}\left|\mathrm{T̂}+{\mathrm{V̂}}_{ee}\right|\Psi_{B}\right\rangle$$ 9

where $\Psi^{\mathrm{LR}}\left[\rho^{B}\right]$ is a ground state of the LR Hamiltonian in eq 7 with the long-range electron interaction $\upsilon_{\mathrm{ee}}^{\mathrm{LR},B}$ . It has been used that electron densities are equal, $\rho_{\Psi^{\mathrm{LR}}}\left(r\right)=\rho^{B}\left(r\right)$ and so are electron-nuclei interaction energies. If the basis set B is sufficiently large and the electron density obtained from $\Psi_{B}$ is close to the exact density, eq 9 immediately leads to an approximate equality between the basis-set incompleteness correction, eq 4, and the multideterminantal SR correlation energy defined in eq 8, namely

10 $$\varepsilon_{B}^{\mathrm{CBS}}\approx \left\langle \Psi \left|\mathrm{T̂}+{\mathrm{V̂}}_{ee}\right|\Psi \right\rangle -\left\langle \Psi_{B}\left|\mathrm{T̂}+{\mathrm{V̂}}_{ee}\right|\Psi_{B}\right\rangle =\left\langle \Psi \left|\mathrm{T̂}+{\mathrm{V̂}}_{ee}\right|\Psi \right\rangle -\left\langle \Psi^{\mathrm{LR}}\left[\rho^{B}\right]\left|\mathrm{T̂}+{\mathrm{V̂}}_{ee}\right|\Psi^{\mathrm{LR}}\left[\rho^{B}\right]\right\rangle \approx E_{c,\mathrm{md}}^{\mathrm{SR}}\left[\rho^{B}\right]$$

where the approximate relations would turn to equalities if $\rho^{B}\left(r\right)=\rho_{\Psi }\left(r\right)$ , i.e. electron density obtained in a basis B were exact.

The equivalence defined in eq 10 is at the heart of the Density-Based Basis-Set Correction. In ref 13 a particular form of the long-range interaction $\upsilon_{\mathrm{ee}}^{\mathrm{LR},B}$ for DBBSC has been proposed. Before we sketch main steps in construction of $\upsilon_{\mathrm{ee}}^{\mathrm{LR},B},$ notice that even if the long-range interaction is such that the assumption in eq 9 is exactly satisfied, the accuracy of the DBBSC will still hinge upon the approximations used for the *E*_*c*,md_^SR^ functional (its exact form is not known) and closeness of the electron density obtained in a given basis set to the exact one. In the first step leading to approximate $\upsilon_{\mathrm{ee}}^{\mathrm{LR},B}$ , the electron–electron Coulomb operator projected onto the basis set B , see the second term in eq 1, has been represented in real space by an effective interaction $W_{\Psi^{B}}\left(r_{1},r_{2}\right)$ assumed as^13^

11 $$W_{\Psi^{B}}\left(r_{1},r_{2}\right)=\frac{\underset{pqrstu}{\sum }g_{pqtu}\Gamma_{pqrs}\varphi_{t}\left(r_{1}\right)\varphi_{u}\left(r_{2}\right)\varphi_{r}\left(r_{1}\right)\varphi_{s}\left(r_{2}\right)}{\rho^{\left(2\right)}\left(r_{1},r_{2}\right)}$$

where the electron pair density function ρ^(2)^(**r**_1_,**r**_2_) is the diagonal part of the two-electron reduced density matrix Γ obtained from the wave function $\Psi^{B}$ . In the second step, $W_{\Psi^{B}}\left(r_{1},r_{2}\right)$ has been mapped on the long-range electronic interaction $\upsilon_{\mathrm{ee}}^{\mathrm{LR},B}$ given in terms of the error function $\upsilon_{\mathrm{ee}}^{\mathrm{LR},B}\left(r_{12}\right)=\mathrm{erf}\left(\mu^{B}r_{12}\right)r_{12}^{-1}$ . The mapping requires that locally $W_{\Psi^{B}}\left(r_{1},r_{2}\right)$ and $\upsilon_{\mathrm{ee}}^{\mathrm{LR},B}\left(r_{12}\right)$ coincide at electron coalescence

$$\underset{r_{1}\to r_{2}}{\lim}W_{\Psi^{B}}\left(r_{1},r_{2}\right)=\upsilon_{ee}^{\mathrm{LR},B}\left(r_{12}\to 0\right)$$ 12

which implies that a basis-set-specific range-separation parameter $\mu^{B}$ acquires position dependence and takes the form

13 $$\mu^{B}\left(r\right)=\frac{\sqrt{\pi }}{2}W_{\Psi^{B}}\left(r,r\right)$$

By construction, $\mu^{B}\left(r\right)$ tends to infinity in the B→CBS limit. Since, by definition, the SR correlation functional vanishes for infinite range-separation parameter, $E_{c,\mathrm{md}}^{\mathrm{SR}}\left[\mu^{B}\to \infty ,\rho^{B}\right]=0$ , one concludes that the basis set correction, eq 10, must vanish in CBS.

## Basis Set Incompleteness-Corrected Correlation Energy

Basis set-specific electron correlation energy is generally defined for a reference wave function $\Psi_{B}^{\mathrm{ref}}$ as a difference between the exact energy in B , given by the full configuration interaction (FCI) wave function $\Psi_{B}^{\mathrm{FCI}}$ , and the reference energy

14 $$E_{\mathrm{corr}}^{B}=\left\langle \Psi_{B}^{\mathrm{FCI}}\left|{Ĥ}_{B}\right|\Psi_{B}^{\mathrm{FCI}}\right\rangle -\left\langle \Psi_{B}^{\mathrm{ref}}\left|{Ĥ}_{B}\right|\Psi_{B}^{\mathrm{ref}}\right\rangle$$

The BSI correction $\varepsilon_{B}^{\mathrm{CBS}}$ , defined in eq 4 with Ψ = Ψ^FCI^ and $\Psi_{B}=\Psi_{B}^{\mathrm{FCI}}$ , recovers the exact energy *E*_0_ = ⟨Ψ^FCI^|*Ĥ*|Ψ^FCI^⟩

15 $$E_{0}=\left\langle \Psi_{B}^{\mathrm{ref}}\left|{Ĥ}_{B}\right|\Psi_{B}^{\mathrm{ref}}\right\rangle +E_{\mathrm{corr}}^{B}+\varepsilon_{B}^{\mathrm{CBS}}$$

Relying on the ideas put forward in ref 13, below we propose a new basis set correction which is obtained together with the electron correlation energy by a modification of the electron interaction operator. In our approach, a single computation recovers $E_{\mathrm{corr}}^{\mathrm{CBS},B}$ —the correlation energy corrected for the BSI error

16 $$E_{\mathrm{corr}}^{\mathrm{CBS},B}=E_{\mathrm{corr}}^{B}+\varepsilon_{B}^{\mathrm{CBS}}$$

As it has been discussed, the operators *Ĥ* ^LR^ and ${Ĥ}_{B}$ lead to short-range electron correlation reduced with respect to full Coulomb electron operator. Thus, it is justified to seek a basis-set specific long-range electron interaction operator, by definition satisfying the short and long-range limits given in eqs 5 and 6, which mimics the Coulomb operator projected onto a finite basis set B . Our main assumption is therefore that for a given basis set, there exists a long-range interaction operator $\upsilon_{\mathrm{ee}}^{\mathrm{LR},B}$ , whose action in the full Hilbert space is equivalent to that of the projected Coulomb operator:

$$\exists \upsilon_{ee}^{\mathrm{LR},B}\;\overset{B}{\underset{pqrs}{\sum }}g_{pqrs}{â}_{p}^{†}{â}_{q}^{†}{â}_{s}{â}_{r}=\overset{\mathrm{CBS}}{\underset{pqrs}{\sum }}g_{pqrs}^{\mathrm{LR},B}{â}_{p}^{†}{â}_{q}^{†}{â}_{s}{â}_{r}$$ 17

where

18 $$g_{pqrs}^{\mathrm{LR},B}=\left\langle pq\left|\upsilon_{ee}^{\mathrm{LR},B}\right|rs\right\rangle$$

­(Coulomb integrals *g*_*pqrs*_ are defined in eq 2). The effective long-range interaction $\upsilon_{\mathrm{ee}}^{\mathrm{LR},B}\left(r_{12}\right)$ , determined by a given basis set B , by definition must be finite at electron coalescence and tend to the Coulomb interaction in the CBS limit:

$$\underset{B\to \mathrm{CBS}}{\lim}\upsilon_{ee}^{\mathrm{LR},B}\left(r_{12}\right)=\frac{1}{r_{12}}$$ 19

Let us introduce a SR interaction operator complementary to $\upsilon_{\mathrm{ee}}^{\mathrm{LR},B}$

20 υeeSR,B(r12)=1r12−υeeLR,B(r12)gpqrsSR,B=gpqrs−gpqrsLR,B

From eqs 5 and 6, it follows that $\upsilon_{\mathrm{ee}}^{\mathrm{SR},B}\left(r_{12}\right)$ is singular at *r*_12_ = 0 and decays faster than *r*_12_^–1^ for large distances. Consider the straightforward relation

21 $$\overset{B}{\underset{pqrs}{\sum }}g_{pqrs}{â}_{p}^{†}{â}_{q}^{†}{â}_{s}{â}_{r}=\overset{\mathrm{CBS}}{\underset{pqrs}{\sum }}g_{pqrs}{â}_{p}^{†}{â}_{q}^{†}{â}_{s}{â}_{r}-\overset{B_{⊥}}{\underset{pqrs}{\sum }}g_{pqrs}{â}_{p}^{†}{â}_{q}^{†}{â}_{s}{â}_{r}$$

where the upper summation in the last term indicates that the pertaining operator acts in a subspace of states orthogonal to those that can be represented in B , i.e. terms for which all spatial parts of spinorbitals *pqrs* belong to B are excluded (i.e., only terms with the indices belonging to a set complementary to B and crossterms are included). Employing this relation in eq 17 yields

22 $$\overset{\mathrm{CBS}}{\underset{pqrs}{\sum }}\left(g_{pqrs}-g_{pqrs}^{\mathrm{LR},B}\right){â}_{p}^{†}{â}_{q}^{†}{â}_{s}{â}_{r}=\overset{B_{⊥}}{\underset{pqrs}{\sum }}g_{pqrs}{â}_{p}^{†}{â}_{q}^{†}{â}_{s}{â}_{r}$$

Differences of the Coulomb and long-range integrals yield complementary short-range integrals, see eq 20, which leads to the equality

23 $$\overset{B_{⊥}}{\underset{pqrs}{\sum }}g_{pqrs}{â}_{p}^{†}{â}_{q}^{†}{â}_{s}{â}_{r}=\overset{\mathrm{CBS}}{\underset{pqrs}{\sum }}g_{pqrs}^{\mathrm{SR},B}{â}_{p}^{†}{â}_{q}^{†}{â}_{s}{â}_{r}$$

Thus, if the effective long-range operator satisfying the condition in eq 17 is found, the incompleteness of the Coulomb electron interaction projected onto a finite basis set B , represented by the left-hand side of the obtained equality, can be captured by a complementary short-range interaction. This equivalence will be of central importance in reducing BSI error of the electron correlation energy.

Assume that a reference wave function $\Psi_{B}^{\mathrm{ref}}$ , found in a finite basis set B is an eigenstate of a zeroth-order Hamiltonian ${Ĥ}_{B}^{\left(0\right)},$ , which includes one- and two-particle operators

24 $${Ĥ}_{B}^{\left(0\right)}=\overset{B}{\underset{pq}{\sum }}h_{pq}^{\left(0\right)}{â}_{p}^{†}{â}_{q}+\frac{1}{2}\overset{B}{\underset{pqrs}{\sum }}g_{pqrs}^{\left(0\right)}{â}_{p}^{†}{â}_{q}^{†}{â}_{s}{â}_{r}$$

Computation of the BSI error-free correlation energy defined in eq 16, which can be written as $E_{\mathrm{corr}}^{\mathrm{CBS},B}=\left\langle \Psi^{\mathrm{FCI}}\left|Ĥ\right|\Psi^{\mathrm{FCI}}\right\rangle -\left\langle \Psi_{B}^{\mathrm{ref}}\left|{Ĥ}_{B}\right|\Psi_{B}^{\mathrm{ref}}\right\rangle$ , is carried out with the exact Hamiltonian, eq 3, of the following form

25 $$Ĥ=\overset{\mathrm{CBS}}{\underset{pq}{\sum }}h_{pq}{â}_{p}^{†}{â}_{q}+\frac{1}{2}\overset{\mathrm{CBS}}{\underset{pqrs}{\sum }}g_{pqrs}{â}_{p}^{†}{â}_{q}^{†}{â}_{s}{â}_{r}$$

The difference between *Ĥ* and a zeroth-order Hamiltonian ${Ĥ}_{B}^{\left(0\right)}$ introduced in eq 24 reads

26 $${Ĥ}^{\prime }=Ĥ-{Ĥ}_{B}^{\left(0\right)}=\overset{B}{\underset{pq}{\sum }}h_{pq}^{\prime }{â}_{p}^{†}{â}_{q}+\frac{1}{2}\overset{B}{\underset{pqrs}{\sum }}g_{pqrs}^{\prime }{â}_{p}^{†}{â}_{q}^{†}{â}_{s}{â}_{r}+\overset{B_{⊥}}{\underset{pq}{\sum }}h_{pq}{â}_{p}^{†}{â}_{q}+\frac{1}{2}\overset{B_{⊥}}{\underset{pqrs}{\sum }}g_{pqrs}{â}_{p}^{†}{â}_{q}^{†}{â}_{s}{â}_{r}$$

where *h*_*pq*_^′^ = *h*_*pq*_ – *h*_*pq*_^(0)^ and *g*_*pqrs*_^′^ = *g*_*pqrs*_ – *g*_*pqrs*_^(0)^ . Assume that the BSI error primarily affects the description of the electron–electron cusp, while the one-electron functions (density and density matrix) are converged with respect to the basis set, which allows us to neglect the $\underset{\mathrm{pq}\in B_{⊥}}{\sum }h_{\mathrm{pq}}{â}_{p}^{†}{â}_{q}$ operator. Note that a similar assumption has been adopted in developing the DBBSC correction. Using eq 23, we obtain

27 $${Ĥ}^{\prime }\approx \overset{B}{\underset{pq}{\sum }}h_{pq}^{\prime }{â}_{p}^{†}{â}_{q}+\frac{1}{2}\overset{B}{\underset{pqrs}{\sum }}g_{pqrs}^{\prime }{â}_{p}^{†}{â}_{q}^{†}{â}_{s}{â}_{r}+\frac{1}{2}\overset{\mathrm{CBS}}{\underset{pqrs}{\sum }}g_{pqrs}^{\mathrm{SR},B}{â}_{p}^{†}{â}_{q}^{†}{â}_{s}{â}_{r}$$

In the following, we assume that the basis set B is sufficiently large for most of the BSI correction to the correlation energy to be recovered by the short-range interaction $\upsilon_{\mathrm{ee}}^{\mathrm{SR},B}$ represented in B . This leads to the final form of the dressed operator, defined within a given basis set

28 $${Ĥ}^{\prime }\approx {Ĥ}_{B}^{\prime }+\frac{1}{2}\overset{B}{\underset{pqrs}{\sum }}g_{pqrs}^{\mathrm{SR},B}{â}_{p}^{†}{â}_{q}^{†}{â}_{s}{â}_{r}$$

which is a central achievement of this work. It shows that BSI correction in the correlation energy calculation can be achieved by modifying the interacting operator ${Ĥ}_{B}^{\prime }$ , used to compute basis-set-specific correlation energy, through the addition of a short-range interaction. In the Supporting Information it is shown that DBBSC, i.e. the basis set incompleteness correction defined in eq 10, could be derived within the perturbation theory based on the perturbing Hamiltonian of the form given above [the last term in eq 28]. This confirms that both methods are based on the same assumptions and both recover BSI entirely within a given basis set B .

## Effective Short-Range Correlation Operator

Our aim is to construct a working form of an effective short-range operator that complements the long-range operator satisfying eq 17. Giner and coauthors^13^ introduced an approximate long-range potential designed to fulfill eq 10. Their potential, $\frac{\mathrm{erf}\left[\mu^{B}\left(r_{1}\right)r_{12}\right]}{r_{12}}$ , incorporates a local dependence of the range-separation parameter which follows from the idea of mapping the Coulomb operator projected onto a basis set B via the long-range interaction represented by the error function, as in eq 12. The complementary SR potential to that proposed in ref 13 takes the form $\frac{1-\mathrm{erf}\left[\mu^{B}\left(r_{1}\right)r_{12}\right]}{r_{12}}$ , and for large $\mu^{B}$ it decays exponentially. Building on the idea of mapping and inspired by the aforementioned approximate construction, we propose a SR potential expressed as

29 $$\upsilon_{ee}^{\mathrm{SR},B}\left(r_{12}\right)=e^{-\mu^{B}\left(r_{1}\right)}\frac{1-\mathrm{erf}\left(r_{12}\right)}{r_{12}}$$

The factorized form—where the position dependence of the local parameter μ­(**r**_**1**_) is separated from the interelectronic distance *r*_12_—is essential for reducing the computational cost of the method. The separation enables the use of an approximate resolution of identity, δ­(**r**_1_–**r**′) ≈ ∑_*t*_φ_*t*_(**r**_1_) φ_*t*_(**r**′), allowing the SR integrals to be approximated as a product of one- and two-electron matrices

30 $$g_{pqrs}^{\mathrm{SR},B}=\left\langle pq\left|\upsilon_{ee}^{\mathrm{SR},B}\left(r_{12}\right)\right|rs\right\rangle \approx \underset{t}{\sum }\left\langle p\left|e^{-\mu^{B}\left(r\right)}\right|t\right\rangle \left\langle tq\left|\frac{1-\mathrm{erf}\left(r_{12}\right)}{r_{12}}\right|rs\right\rangle$$

Without the RI approximation, evaluating the μ–dependent SR two-electron integrals would require a six-dimensional numerical integration, which is computationally prohibitive. In practical calculations, the matrix $g^{\mathrm{SR},B}$ of integrals is symmetrized, i.e. one uses $g_{\mathrm{pqrs}}^{\mathrm{SR},B}=\frac{1}{4}\left(g_{\mathrm{p̃}\mathrm{qrs}}^{\mathrm{SR},B}+g_{p\mathrm{q̃}\mathrm{rs}}^{\mathrm{SR},B}+g_{\mathrm{pq}\mathrm{r̃}s}^{\mathrm{SR},B}+g_{\mathrm{pqr}\mathrm{s̃}}^{\mathrm{SR},B}\right)$ , where the tilded index is the index transformed by the matrix of the operator $e^{-\mu^{B}\left(r\right)}$ , to preserve the symmetry of the two-electron integrals used in actual calculations.

To summarize, we propose to construct the modified Hamiltonian *Ĥ*′, defined in eq 28, using the effective short-range interaction introduced in eq 30. It depends on the local range-separation parameter $\mu^{B}\left(r\right)$ , defined analogously to that in the DBBSC method [see eqs 11 and 13]. By design, $\mu^{B}$ increases with the basis set size and tends to infinity in the complete basis set limit. As a result, the effective short-range interaction is suppressed with the extension of the basis set, vanishing in this limit,

$$\forall_{pqrs}\underset{B\to \mathrm{CBS}}{\lim}g_{pqrs}^{\mathrm{SR},B}=0$$ 31

This ensures that the correlation energy computed with the modified Hamiltonian correctly converges to the CBS limit. Notice also that, due to the locality of the $\mu^{B}$ function, the proposed Hamiltonian *Ĥ*′ leads to size-consistent BSI-corrected correlation energies. This can be concluded immediately by considering a dimer AB consisting of two fragments A and B. In the dissociation limit, the range-separation function of the dimer separates into monomer components,^19^ i.e. $\underset{R_{\mathrm{AB}}\to \infty }{\lim}\mu_{\mathrm{AB}}^{B}\left(r\right)=\mu_{X}^{B}\left(r\right)$ for **r** ∈X, where $\mu_{X}^{B}$ (X = A,B) and $\mu_{\mathrm{AB}}^{B}$ are range-separation functions of isolated fragments and the dimer, respectively. Consequently, the effective SR interaction is separable and

$$\forall_{pqrs\in X}\underset{R_{\mathrm{AB}}\to \infty }{\lim}g_{pqrs}^{\mathrm{SR},B}\left(\mu_{\mathrm{AB}}^{B}\right)=g_{pqrs}^{\mathrm{SR},B}\left(\mu_{X}^{B}\right),X=A,B$$ 32

where orbitals in the dissociation limit are localized on one of the monomers, *pqrs*∈X denotes that all orbitals *pqrs* are localized on X. Thus, self-consistent correlation energy methods will retain this property if the Hamiltonian is modified by adding SR effective interaction integrals.

The proposed framework is generally applicable to both single- and multireference correlation energy calculations. For a given correlation energy method assuming partitioning the electronic Hamiltonian into the reference, *Ĥ* ^(0)^, Hamiltonian and the complementary part *Ĥ* ^′^, the following additional steps are involved in obtaining correlation energy with the BSI error reduced: (i) computation of the local range-separation function and long-range two-electron integrals with the erf(*r*_12_)/*r*_12_ operator, (ii) computation of the effective short-range integrals, $g_{\mathrm{pqrs}}^{\mathrm{SR},B}$ , defined in eq 30, (iii) replacing Coulomb integrals, *g*_*pqrs*_, with a sum $g_{\mathrm{pqrs}}+g_{\mathrm{pqrs}}^{\mathrm{SR},B}$ in the two-electron part of the Hamiltonian *Ĥ* ^′^.

In this work, we apply the proposed method to remove the BSI errors of multireference correlation energies corresponding to complete active space (CAS) wave functions. The correlation energy is obtained by the linearized adiabatic connection (AC0) method^28–33^ and the n-electron valence state second-order perturbation theory (NEVPT2).^34–38^ In both methods, the ${Ĥ}_{B}^{\left(0\right)}$ operator is in the form of the Dyall’s Hamiltonian.^33,39^ To account for the BSI error, the perturbing Hamiltonian is modified by adding an effective short-range operator, as shown in eq 28. The resulting AC0 and NEVPT2 correlation energies are denoted by AC0-CBS­[H] and NEVPT2-CBS­[H], respectively. In the proposed framework, the total energy still contains an uncorrected BSI error in the reference (CASSCF) energy. This error could be reduced by employing an auxiliary basis set and computing the complementary auxiliary basis set (CABS) single-excitation correction, as is typically done in multireference F12 methods.^7^ Since this work focuses only on the BSI error in the correlation energy, the CABS correction is not considered here.

## Computational Details

Computation of the AC0 and AC0-CBS­[H] energies requires CASSCF 1- and 2-electron reduced density matrices, which have been obtained from the Molpro^40^ program. The calculations of the AC0 and AC0-CBS­[H] correlation energies have been carried out using GammCor^41^ program. For AC0-CBS­[H], first the symmetrized short-range integrals, eq 30, are computed with the local range-separated parameter $\mu^{B}\left(r\right)$ constructed according to eq 13 and eq 11. We have reduced the cost of computing the $\mu^{B}\left(r\right)$ function by introducing Cholesky decomposition of Coulomb integrals in the calculation of the effective interaction at electron coalescence, $W_{\Psi^{B}}\left(r,r\right)$ , defined in eq 11. The most computationally demanding steps in the calculation scale with the fifth power with the size of the system. Specifically, the leading cost terms scale as *n*_*a*_^4^ *n*_grid_ and (*n*_*i*_+*n*_*a*_)^2^ *n*_Chol_ *n*_grid_, where *n*_*i*_ and *n*_*a*_ refer to the number of inactive and active orbitals, respectively, *n*_Chol_ is the number of Cholesky vectors, and *n*_grid_ is the number of grid points used in the DFT quadrature (for details, see the Appendix of ref 42). Notice that our algorithm for constructing $\mu^{B}\left(r\right)$ could be seen as an extension of that proposed in ref 17, which is based on density fitting. The latter algorithm has been developed for single-determinantal wave functions, while our scheme is designed to be used with CASSCF two-electron density matrices. Matrix elements of the $\exp\left[-\mu^{B}\left(r\right)\right]$ operator follow from the numerical integration. Molecular electron integrals were obtained with the gammcor-integrals^43^ library. NEVPT2 and NEVPT2-CBS­[H] calculations were carried out with the PySCF code^44^ with additional custom subroutines.

The AC0-CBS­[H] and NEVPT2-CBS­[H] results are compared with the AC0 and NEVPT2 energies corrected for the BSI error by adding the DBBSC correction given in eq 10, computed using the PBE-based short-range functional from ref 15 and on-top pair density from CASSCF wave functions. The employed DBBSC variant corresponds to SU-PBE-OT from ref 19 without freezing core orbitals. The resulting energies are be denoted as AC0-DBBSC and NEVPT2-DBBSC. The DBBSC correction was implemented in GammCor.^41^

The following active spaces were used in CASSCF computations for molecules: N_2_ – CAS­(8,10), H_2_O – CAS­(8,6), O_2_ – CAS­(8,6), F_2_ – CAS­(14,8), CH_2_ – CAS­(6,6). Equilibrium geometries adopted in tests are N_2_ – *R*_NN_ = 2.070 au; H_2_O – *R*_OH_ = 1.809 au, ∠HOH = 104.5°; O_2_ – *R*_OO_ = 2.282 au; F_2_ – *R*_FF_ = 2.730 au. Dissociation energies were computed for the following stretched-bond geometries: N_2_ – *R*_NN_ = 10.000 au; H_2_O – *R*_OH_ = 9.500 au; O_2_ – *R*_OO_ = 10.000 au, and F_2_ – *R*_FF_ = 9.500 au. In linearization of the CH_2_ molecule the HCH angle has changed from ∠HCH = 102.3° and ∠HCH = 133.0° for singlet and triplet states, respectively to ∠HCH = 180°. The bond length has been fixed at *R*_CH_ = 2.09 au. Calculations were carried out using Dunning’s cc-pV*X*Z basis sets.^45^

## Results

First, we investigate the accuracy of the BSI-corrected correlation energy $E_{\mathrm{corr}}^{\mathrm{CBS},B}$ for the helium atom using Hartree–Fock (HF), CAS­(2,5)­SCF and CAS­(2,14)­SCF reference wave functions. The correlation energy in a given basis set, $E_{\mathrm{corr}}^{B}$ , is computed according to eq 14. The $E_{\mathrm{corr}}^{\mathrm{CBS},B}$ correlation energy, defined in eq 16, is found following the approximate method proposed in this work. Thus, it consists in taking a difference of the corrected FCI and the reference energies $E_{\mathrm{corr}}^{\mathrm{CBS},B}=\left\langle \Psi_{B}^{\mathrm{FCI}}\left|Ĥ\left[B,\Psi_{B}^{\mathrm{ref}}\right]\right|\Psi_{B}^{\mathrm{FCI}}\right\rangle -\left\langle \Psi_{B}^{\mathrm{ref}}\left|{Ĥ}_{B}\right|\Psi_{B}^{\mathrm{ref}}\right\rangle$ where $\Psi_{B}^{\mathrm{FCI}}$ is a full CI function that diagonalizes the following Hamiltonian

33 $$Ĥ\left[B,\Psi_{B}^{\mathrm{ref}}\right]={Ĥ}_{B}^{\left(0\right)}+{Ĥ}^{\prime }={Ĥ}_{B}+\frac{1}{2}\overset{B}{\underset{pqrs}{\sum }}g_{pqrs}^{\mathrm{SR},B}{â}_{p}^{†}{â}_{q}^{†}{â}_{s}{â}_{r}$$

Clearly, the total energy, uncorrected for basis set incompleteness, computed as the sum of the reference energy $E_{B}^{\mathrm{ref}}=$ $\left\langle \Psi_{B}^{\mathrm{ref}}\left|{Ĥ}_{B}\right|\Psi_{B}^{\mathrm{ref}}\right\rangle$ $\mathrm{and}\;E_{corr}^{B},$ i.e. $E_{B}^{\mathrm{ref}}+E_{\mathrm{corr}}^{B}=$ $\left\langle \Psi_{B}^{\mathrm{FCI}}\left|{Ĥ}_{B}\right|\Psi_{B}^{\mathrm{FCI}}\right\rangle$ , does not depend on the reference function. Contrary to that, the energy corrected for BSI, $E_{B}^{\mathrm{ref}}+E_{\mathrm{corr}}^{\mathrm{CBS},B}=\left\langle \Psi_{B}^{\mathrm{FCI}}\left|Ĥ\left[B,\Psi_{B}^{\mathrm{ref}}\right]\right|\Psi_{B}^{\mathrm{FCI}}\right\rangle$ , will in practice depend on both the basis set and the reference wave function. Notice that if the reference wave function is equal to the FCI function in a given basis set, then $E_{\mathrm{corr}}^{B}=0$ , but the corrected correlation energy $E_{\mathrm{corr}}^{\mathrm{CBS},B}$ will be different from zero.

In Figure 1, we present electron pair densities obtained from the FCI wave function using both the unmodified Hamiltonian, ${Ĥ}_{B}$ , and the Hamiltonian $Ĥ\left[B,\Psi_{B}^{\mathrm{ref}}\right]$ , where $\Psi_{B}^{\mathrm{ref}}$ is a HF reference. For a given basis set, one observes deepening of the pair function corresponding to $Ĥ\left[B,\Psi_{B}^{\mathrm{ref}}\right]$ in the electron coalescence region, compared to that obtained with the unmodified Hamiltonian. This confirms that the effective short-range operator deepens the Coulomb hole around the position of a reference electron. Its effect is therefore analogous to increasing the basis set size—short-range electron correlation is strengthened.

**Figure 1:**
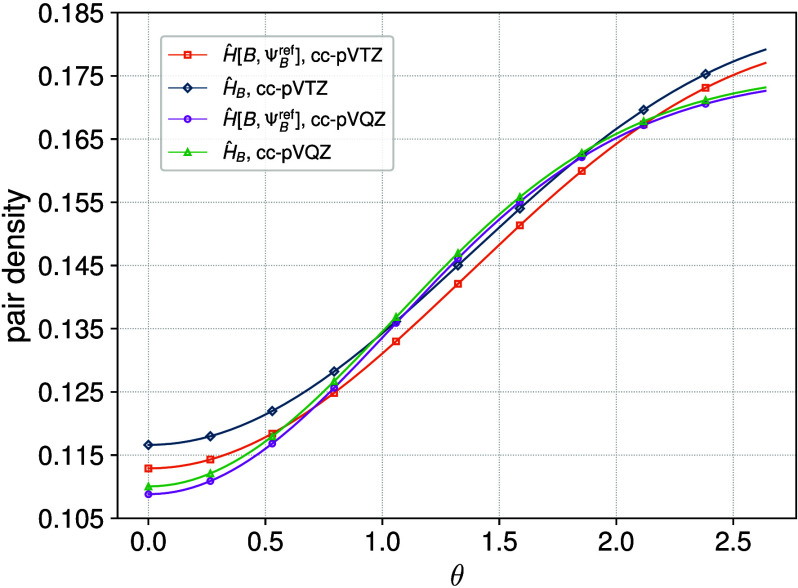
Electron pair density on a sphere of radius 0.5 au for the helium atom as a function of the angle between the position vectors of the two electrons. ${Ĥ}_{B}$ and $Ĥ\left[B,\Psi_{B}^{\mathrm{ref}}\right]$ correspond to the unmodified Hamiltonian in a given basis set and the Hamiltonian modified according to eq 33, respectively.

In Table 1 we report BSI corrections to the correlation energies, $\varepsilon_{B}^{\mathrm{CBS}}=E_{\mathrm{corr}}^{\mathrm{CBS},B}-E_{\mathrm{corr}}^{B}$ , eq 16, for the helium atom obtained using the proposed approximate scheme, denoted as $\varepsilon_{B}^{\mathrm{CBS}\left[H\right]}$ . They are compared with DBBSC results and the exact values. The latter are computed following eq 4 as $E_{\mathrm{exact}}-E_{B}^{\mathrm{FCI}}$ , where $E_{B}^{\mathrm{FCI}}=\left\langle \Psi_{B}^{\mathrm{FCI}}\left|{Ĥ}_{B}\right|\Psi_{B}^{\mathrm{FCI}}\right\rangle$ is the FCI energy in a given basis set, and the exact energy for helium equal to – 2.903724 Ha is taken from ref 46.

**Table 1:** Basis Set Incompleteness Corrections to Correlation Energies of the Helium Atom Obtained Using HF, CAS­(2,5)­SCF, and CAS­(2,14)­SCF Reference Wavefunctions^a^

	HF		CAS(2,5)SCF		CAS(2,14)SCF		
cc-pVXZ	$\epsilon_{B}^{\mathrm{CBS}\left[H\right]}$	$\epsilon_{B}^{\mathrm{DBBSC}}$	$\epsilon_{B}^{\mathrm{CBS}\left[H\right]}$	$\epsilon_{B}^{\mathrm{DBBSC}}$	$\epsilon_{B}^{\mathrm{CBS}\left[H\right]}$	$\epsilon_{B}^{\mathrm{DBBSC}}$	exact
DZ	–9.0	–12.3	–8.3	–9.5			–16.1
TZ	–3.9	–5.1	–3.7	–3.7	–3.3	–3.6	–3.5
QZ	–1.9	–2.3	–1.8	–1.6	–1.5	–1.8	–1.3
5Z	–1.1	–1.3	–1.0	–0.9	–0.8	–1.0	–0.6
6Z	–0.7	–0.8	–0.6	–0.5	–0.6	–0.5	–0.3

a “Exact” correction computed as $E_{\mathrm{exact}}-E_{B}^{\mathrm{FCI}}$ , where *E*_exact_ is taken from ref 46. Energy unit is mHa.

The poor performance of $\epsilon_{B}^{\mathrm{CBS}\left[H\right]}$ in the cc-pVDZ basis could be attributed to the approximation applied in going from eq 27 to eq 28, i.e. projecting the operator $\upsilon_{\mathrm{ee}}^{\mathrm{SR},B}$ onto the Hilbert space spanned by B . The DBBSC correction is only slightly more accurate in this basis set. Expanding the basis set already to cc-pVTZ leads to a dramatic improvement in accuracy. The CBS­[H] correction obtained for the CASSCF references agrees with the exact value to within 0.2 mHa. For larger basis sets, the error remains within the submillihartree regime and reaches at most 0.5 mHa. A comparison with the DBBSC correction shows that both methods perform equally well on average. For a given basis set, both CBS­[H] and DBBSC corrections are nearly independent of the reference CASSCF wave functions, as they should. When applied with the HF reference, CBS­[H] and DBBSC also perform on par, with the exception of the cc-pVTZ basis, where the CBS­[H] error is 1 mHa larger than that of DBBSC. The HF-based errors are larger than for CASSCF wave functions, exceeding 0.5 mHa.

The CBS­[H] method was validated for absolute energies at equilibrium geometries and for relative energies on a set of representative molecules. Figure 2 presents errors in the absolute energies computed with respect to the estimated benchmark values (see also Figure S1, Tables S1 and S2 in the Supporting Information). Benchmark results are calculated using a two-point extrapolation scheme^47^ from cc-pV5Z and cc-pV6Z values of the uncorrected correlation energy (AC0 or NEVPT2). In all cases, the CBS­[H]-corrected AC0 and NEVPT2 correlation energies computed in a basis set with cardinal number *X* (starting from X = 3) approach the uncorrected values obtained with (*X*+1) basis set. Notably, the CBS­[H] method is equally effective for AC0 and NEVPT2: the improvement of convergence with the basis set size is similar for both methods. The DBBSC correction reduces the correlation energy error to a few mHa already in a triple-ζ basis. However, in QZ and 5Z basis sets, the DBBSC model overcorrects, and the energy error becomes negative. The CBS­[H] does not exhibit this error.

**Figure 2:**
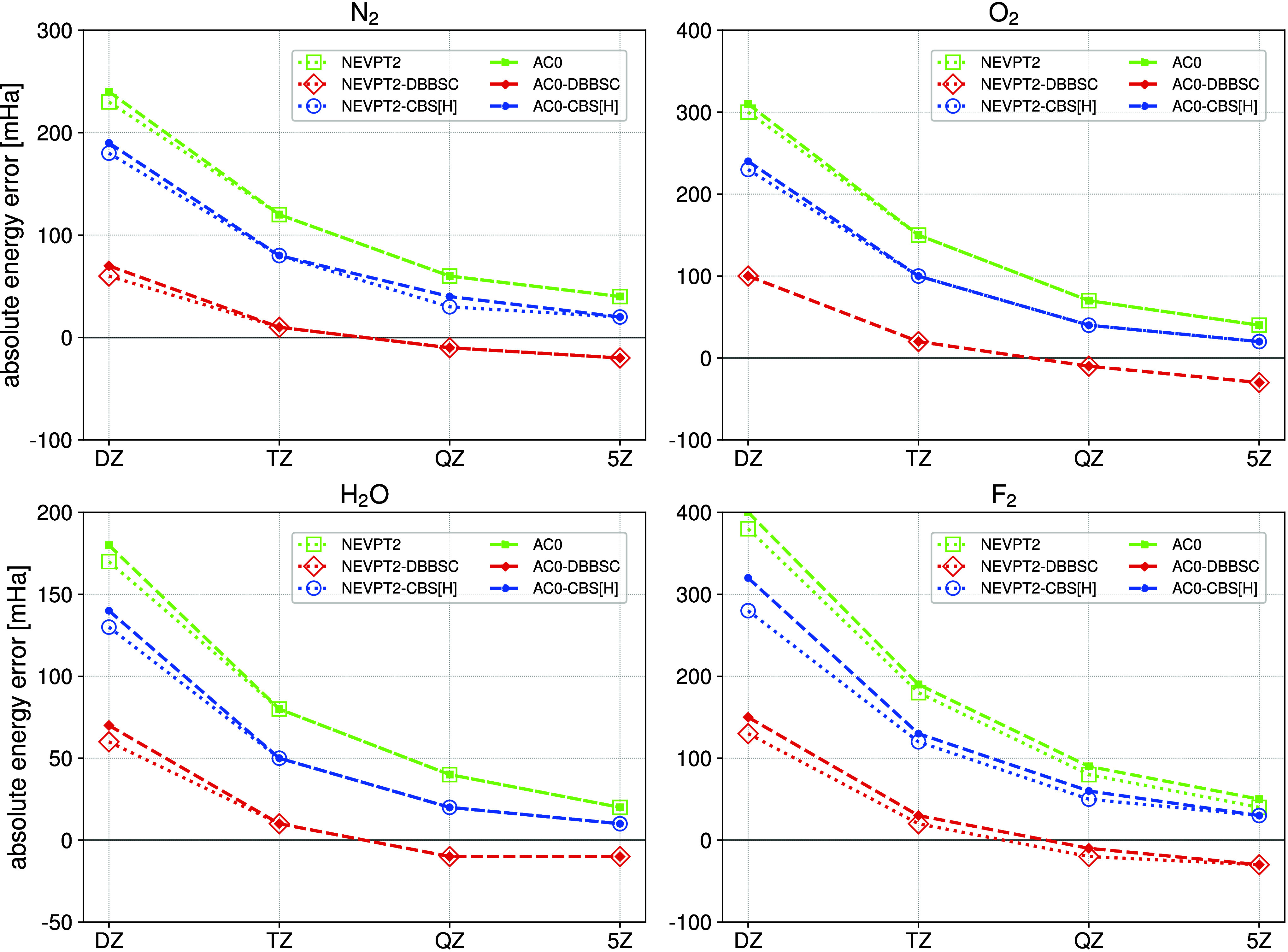
Absolute energy errors as a function of the cardinal number *X* for N_2_, O_2_, H_2_O, and F_2_ molecules in equilibrium geometries.

In Figures 3–4 and Table 2, we present reduction of the BSI error in relative energies as a function of the cardinal number *X*. For the nitrogen molecule, the AC0-CBS­[H] dissociation energy in the TZ basis set recovers the uncorrected AC0 energy in the 5Z basis set to within 1 mHa. The NEVPT2-CBS­[H] error in the TZ basis is larger and approaches that of the uncorrected NEVPT2 in the QZ basis. For the remaining molecules, the performance of AC0-CBS­[H] is excellent and the dissociation energy error drops below 1 mHa already in the TZ basis set. Combined with NEVTP2, CBS­[H] performs slightly worse, with errors for the O_2_ and F_2_ dimers exceeding 1 mHa at the TZ level. Still, the CBS­[H] correction gains two cardinal numbers in accuracy relative to the uncorrected NEVPT2 energy. Similar performance is observed for CBS­[H] in the case of the linearization energy barrier of CH_2_ in both singlet and triplet states, as well as for the singlet–triplet (ST) gap, cf. the lower entries of Table 2 and Figure 4. In the TZ basis set, the CBS­[H]-corrected correlation energies closely match the uncorrected AC0 or NEVPT2 results obtained in the 5Z basis set. On average, CBS­[H] and DBBSC offer similar accuracy for relative correlation energies in basis sets larger than DZ. In the cc-pVTZ basis set, the DBBSC-corrected relative energies remain within an error margin of 1.5 mHa for all molecules. In CBS­[H] calculations in the same basis, the nitrogen molecule is a clear outlier. On the other hand, for O_2_ and F_2_, AC0-CBS­[H] achieves 0.5 mHa accuracy at the TZ level, whereas DBBSC errors exceed 1 mHa.

**Figure 3:**
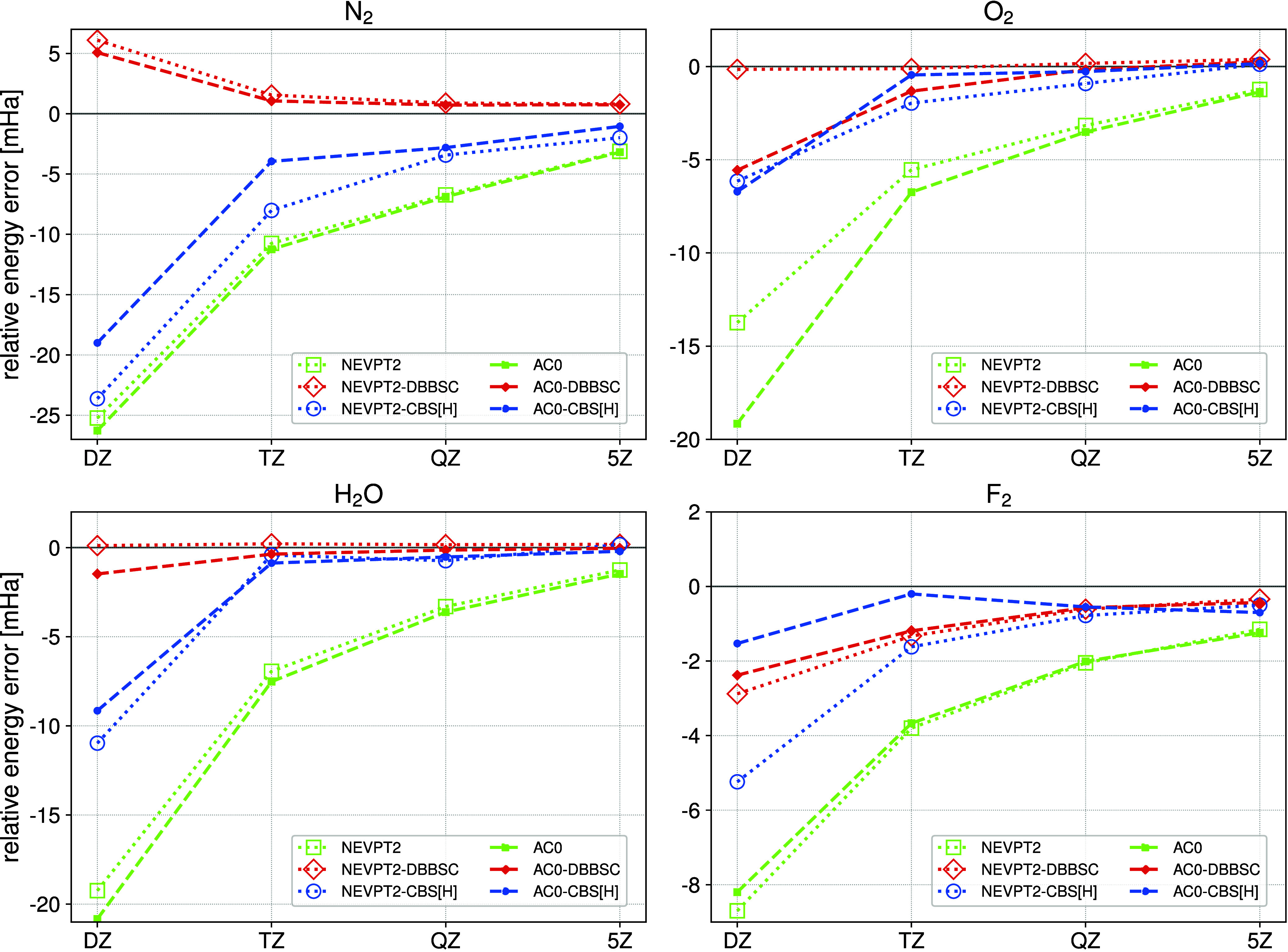
Errors of dissociation energies as a function of the cardinal number *X* for N_2_, O_2_, H_2_O, and F_2_ molecules.

**Figure 4:**
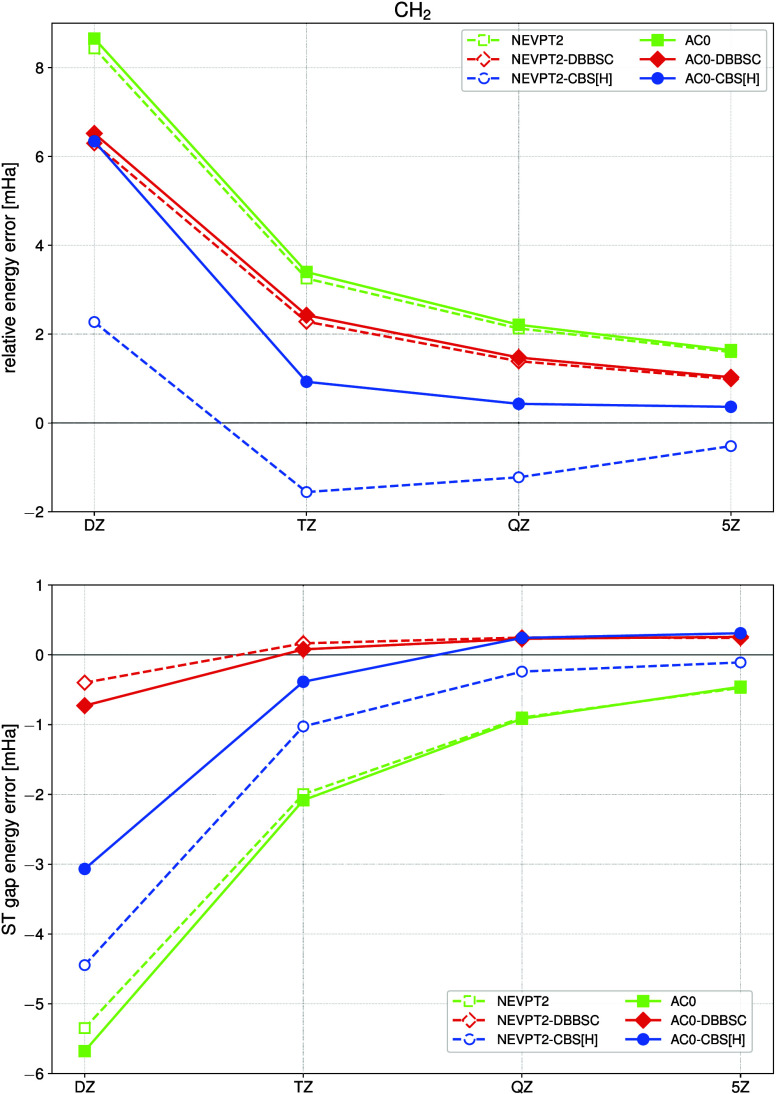
Errors in relative energies for the CH_2_ molecule as a function of the cardinal number *X*. Upper panel: Energy barrier for linearization of the molecule in the singlet state (the HCH angle changes from 102.3° to 180.0°, *R*_CH_ = 2.09 au). Lower panel: ST energy gap for a molecule at equilibrium geometry (∠HCH = 102.3°, *R*_CH_ = 2.09 au).

**Table 2:** Errors in Relative Energies (Dissociation Energies for N_2_, H_2_O, O_2_, and F_2_ Molecules and Linearization Barriers for CH_2_ in Singlet and Triplet States) as a Function of the Cardinal Number *X* ^a^

system	basis	NEVPT2	NEVPT2-DBBSC	NEVPT2-CBS[H]	AC0	AC0-DBBSC	AC0-CBS[H]
N_2_	DZ	–25.23	6.10	–23.63	–26.27	5.07	–19.00
	TZ	–10.75	1.55	–8.02	–11.24	1.06	–3.95
	QZ	–6.73	0.88	–3.43	–6.89	0.72	–2.81
	5Z	–3.12	0.81	–1.99	–3.19	0.74	–1.05
H_2_O	DZ	–19.24	0.16	–10.97	–20.83	–1.48	–9.15
	TZ	–6.94	0.23	–0.42	–7.52	–0.37	–0.87
	QZ	–3.31	0.19	–0.74	–3.62	–0.14	–0.53
	5Z	–1.26	0.19	0.17	–1.48	–0.04	–0.21
O_2_	DZ	–13.74	–0.15	–6.15	–19.16	–5.56	–6.71
	TZ	–5.54	–0.11	–1.96	–6.74	–1.32	–0.45
	QZ	–3.16	0.17	–0.91	–3.51	–0.17	–0.27
	5Z	–1.23	0.39	0.12	–1.36	0.26	0.16
F_2_	DZ	–8.70	–2.88	–5.24	–8.20	–2.38	–1.53
	TZ	–3.80	–1.33	–1.62	–3.67	–1.19	–0.20
	QZ	–2.05	–0.61	–0.78	–2.01	–0.57	–0.55
	5Z	–1.15	–0.34	–0.50	–1.24	–0.43	–0.70
CH_2_(^1^ *A*_1_)	DZ	8.44	6.30	2.27	8.66	6.52	6.34
	TZ	3.25	2.28	–1.56	3.40	2.43	0.93
	QZ	2.13	1.39	–1.22	2.21	1.47	0.43
	5Z	1.60	0.99	–0.52	1.64	1.03	0.36
CH_2_(^3^ *B*_1_)	DZ	2.03	2.12	0.11	1.96	2.06	1.76
	TZ	0.70	0.69	–0.98	0.69	0.68	0.31
	QZ	0.71	0.60	0.01	0.71	0.60	0.44
	5Z	0.71	0.51	0.13	0.70	0.51	0.51
MUE	TZ	5.16	1.03	2.43	5.54	1.17	1.12

a Energy unit is mHa.

In the DZ basis set, CBS­[H] errors exceed 5 mHa, except for F_2_ and CH_2_. While this is still an improvement over the uncorrected results, DBBSC in the same basis is clearly more accurate, with AC0-DBBSC errors ranging from – 1.5 to 5.6 mHa. The superior performance of DBBSC over CBS­[H] stems from a different construction of these two methods. By design, DBBSC accounts for the BSI error in both the reference and the correlation energies while CBS­[H], in its current formulation, corrects only the correlation energy. (Notice that it has been proposed to apply the CABS single excitation correction for the reference energy in addition to the DBBSC correction, to further reduce the BSI error.^17^) Moreover, in a basis set as small as DZ, the resolution of identity assumption in eq 30 underlying CBS­[H] may be violated. To investigate this further, we carried out computations in the cc-pVXZ-F12 basis set,^48^ optimized for explicitly correlated F12 methods. Results collected in the Supporting Information show that the errors of relative CBS­[H]/cc-pVDZ-F12 energies are, on average, smaller than in the standard cc-pVDZ basis. Notably, for NEVPT2-CBS­[H], they approach the values of uncorrected NEVPT2 in cc-pVTZ-F12 basis.

## Conclusions

We have proposed the CBS­[H] method to reduce the basis set incompleteness error in the correlation energy. It is based on the assumption that a local mapping exists between the Coulomb electron interaction projected onto a finite basis set and a long-range interaction described by the error function with a local range-separation parameter. If this mapping holds, the corresponding short-range interaction is equivalent to the Coulomb interaction projected onto the space complementary to the chosen basis set. Building on the construction of the long-range interaction with a local range-separation parameter introduced in ref 13, we have proposed an approximate form for the complementary interaction. The CBS­[H] method modifies the Hamiltonian used to compute the correlation energy by adding an effective short-range interaction operator. As a result, the correlation energy corrected for BSI error is obtained in a single calculation.

Using the helium atom as a test case, we demonstrated that CBS­[H] recovers the correlation energy with an accuracy better than 0.5 mHa in a triple-ζ basis set for both HF and CASSCF reference wave functions. We applied CBS­[H] with the multireference AC0 and NEVPT2 methods to compute correlation energies for a set of representative small molecules. CBS­[H] achieves an accuracy gain in relative energies of approximately two cardinal numbers with respect to the basis set size, starting from a triple-ζ basis. This is comparable to the performance of the DBBSC correction.^13^

It is worth emphasizing that CBS­[H] reduces the BSI error at a competitive cost compared to other non-DFT-based approaches, e.g. explicitly correlated F12 or transcorrelated Hamiltonian methods. Unlike these methods, CBS­[H] requires no auxiliary basis sets and involves only two-electron integrals. Its computational cost is comparable to that of DBBSC, as both methods rely on evaluating a local range-separation parameter. With the Cholesky decomposition of two-electron integrals, employed in construction of the range-separation parameter, the CBS­[H] scaling is reduced to below the fifth power of the system size. This ensures that the CBS­[H] approximation does not introduce any additional overhead relative to the overall computational cost of correlation energy calculations.

A potential advantage of the method over DBBSC is its direct applicability to response properties, as the underlying dressing of the Hamiltonian with a short-range effective interaction is, in principle, universally valid. Future work will further explore broader applications of CBS­[H].

## Supplementary Material



## Notes

Derivation of DBSSC from the perturbation theory, absolute energies in cc-pVXZ basis sets, and absolute and relative energies in cc-pVXZ-F12 basis sets (PDF)

## References

[1] Werner H.-J., Adler T. B., Manby F. R. (2007). General
orbital invariant MP2-F12
theory. J. Chem. Phys..

[2] Fliegl H., Klopper W., Hättig C. (2005). Coupled-cluster theory with simplified
linear-r12 corrections: The CCSD (R12) model. J. Chem. Phys..

[3] Werner H.-J., Knizia G., Manby F. R. (2011). Explicitly
correlated coupled cluster
methods with pair-specific geminals. Mol. Phys..

[4] Kong L., Bischoff F. A., Valeev E. F. (2012). Explicitly
correlated R12/F12 methods
for electronic structure. Chem. Rev..

[5] Shiozaki T., Werner H.-J. (2013). Multireference explicitly
correlated F12 theories. Mol. Phys..

[6] Shiozaki T., Werner H.-J. (2010). Communication: Second-order
multireference perturbation
theory with explicit correlation: CASPT2-F12. J. Chem. Phys..

[7] Guo Y., Sivalingam K., Valeev E. F., Neese F. (2017). Explicitly correlated
N-electron valence state perturbation theory (NEVPT2-F12). J. Chem. Phys..

[8] Boys S. F., Handy N. C. (1969). The determination
of energies and wavefunctions with
full electronic correlation. Proc. R. Soc. Lond.
A.

[9] Ten-no S. (2000). A feasible
transcorrelated method for treating electronic cusps using a frozen
Gaussian geminal. Chem. Phys. Lett..

[10] Yanai T., Shiozaki T. (2012). Canonical transcorrelated
theory with projected Slater-type
geminals. J. Chem. Phys..

[11] Cohen A. J., Luo H., Guther K., Dobrautz W., Tew D. P., Alavi A. (2019). Similarity
transformation of the electronic Schrödinger equation via Jastrow
factorization. J. Chem. Phys..

[12] Giner E. (2021). A new form
of transcorrelated Hamiltonian inspired by range-separated DFT. J. Chem. Phys..

[13] Giner E., Pradines B., Ferté A., Assaraf R., Savin A., Toulouse J. (2018). Curing basis-set convergence
of wave-function theory
using density-functional theory: A systematically improvable approach. J. Chem. Phys..

[14] Toulouse J., Gori-Giorgi P., Savin A. (2005). A short-range correlation energy
density functional with multi-determinantal reference. Theor. Chem. Acc..

[15] Ferté A., Giner E., Toulouse J. (2019). Range-separated multideterminant
density-functional theory with a short-range correlation functional
of the on-top pair density. J. Chem. Phys..

[16] Loos P.-F., Pradines B., Scemama A., Toulouse J., Giner E. (2019). A Density-Based
Basis-Set Correction for Wave Function Theory. J. Phys. Chem. Lett..

[17] Heßelmann A., Giner E., Reinhardt P., Knowles P. J., Werner H.-J., Toulouse J. (2024). A density-fitting implementation
of the density-based
basis-set correction method. J. Comput. Chem..

[18] Giner E., Scemama A., Toulouse J., Loos P.-F. (2019). Chemically accurate
excitation energies with small basis sets. J.
Chem. Phys..

[19] Giner E., Scemama A., Loos P.-F., Toulouse J. (2020). A basis-set error correction
based on density-functional theory for strongly correlated molecular
systems. J. Chem. Phys..

[20] Mester D., Kállay M. (2023). Basis set limit of CCSD (T) energies: explicit correlation
versus density-based basis-set correction. J.
Chem. Theory Comput..

[21] Mester D., Nagy P. R., Kállay M. (2024). Basis-set
limit CCSD (T) energies
for large molecules with local natural orbitals and reduced-scaling
basis-set corrections. J. Chem. Theory Comput..

[22] Savin, A. On degeneracy, near–degenaracy and density functional theory. In Recent Developments and Applications of Modern Density Functional Theory. Seminario, J. M. , Ed.; Elsevier: Amsterdam, The NEtherlands, 1996; pp 327–357.

[23] Stoll, H. , Savin, A. Density Functionals for Correlation Energies of Atoms and Molecules. In Density Functional Methods in Physics. Dreizler, R. , da Providência, J. , Eds.; Plenum, New York, NY, 1985; pp 177–207.

[24] Toulouse J., Colonna F., Savin A. (2004). Long-range-short-range
separation
of the electron-electron interaction in density-functional theory. Phys. Rev. A.

[25] Pollet R., Savin A., Leininger T., Stoll H. (2002). Combining multideterminantal
wave functions with density functionals to handle near-degeneracy
in atoms and molecules. J. Chem. Phys..

[26] Pernal K., Hapka M. (2022). Range-separated multiconfigurational
density functional theory methods. WIREs Comput.
Mol. Sci..

[27] Gori-Giorgi P., Savin A. (2006). Properties of short-range
and long-range correlation energy density
functionals from electron-electron coalescence. Phys. Rev. A.

[28] Pernal K. (2018). Electron Correlation
from the Adiabatic Connection for Multireference Wave Functions. Phys. Rev. Lett..

[29] Pastorczak E., Pernal K. (2018). Correlation Energy from the Adiabatic Connection Formalism
for Complete Active Space Wave Functions. J.
Chem. Theory Comput..

[30] Pastorczak E., Pernal K. (2018). Electronic Excited States from the
Adiabatic-Connection
Formalism with Complete Active Space Wave Functions. J. Phys, Chem. Lett..

[31] Pastorczak E., Hapka M., Veis L., Pernal K. (2019). Capturing
the Dynamic
Correlation for Arbitrary Spin-Symmetry CASSCF Reference with Adiabatic
Connection Approaches: Insights into the Electronic Structure of the
Tetramethyleneethane Diradical. J. Phys. Chem.
Lett..

[32] Beran P., Matoušek M., Hapka M., Pernal K., Veis L. (2021). Density matrix
renormalization group with dynamical correlation via adiabatic connection. J. Chem. Theory Comput..

[33] Guo Y., Pernal K. (2024). Spinless formulation of linearized adiabatic connection
approximation and its comparison with second order N-electron valence
state perturbation theory. Faraday Discuss..

[34] Angeli C., Cimiraglia R., Evangelisti S., Leininger T., Malrieu J.-P. (2001). Introduction of
n-electron valence states for multireference
perturbation theory. J. Chem. Phys..

[35] Angeli C., Cimiraglia R., Malrieu J.-P. (2001). N-electron valence state perturbation
theory: a fast implementation of the strongly contracted variant. Chem. Phys. Lett..

[36] Angeli C., Cimiraglia R., Malrieu J.-P. (2002). n-electron valence
state perturbation
theory: A spinless formulation and an efficient implementation of
the strongly contracted and of the partially contracted variants. J. Chem. Phys..

[37] Guo Y., Sivalingam K., Neese F. (2021). Approximations of density matrices
in N-electron valence state second-order perturbation theory (NEVPT2).
I. Revisiting the NEVPT2 construction. J. Chem.
Phys..

[38] Guo Y., Sivalingam K., Kollmar C., Neese F. (2021). Approximations of density
matrices in N-electron valence state second-order perturbation theory
(NEVPT2). II. The full rank NEVPT2 (FR-NEVPT2) formulation. J. Chem. Phys..

[39] Dyall K. G. (1995). The choice
of a zeroth-order Hamiltonian for second-order perturbation theory
with a complete active space self-consistent-field reference function. J. Chem. Phys..

[40] Werner H.-J., Knowles P. J., Knizia G., Manby F. R., Schütz M. (2012). Molpro: a
general-purpose quantum chemistry program package. WIREs Comput. Mol. Sci..

[41] Pernal, K. ; Hapka, M. ; Przybytek, M. ; Modrzejewski, M. ; Sokół, A. ; Tucholska, A. GammCor code. GitHub, 2020. https://github.com/pernalk/GAMMCOR (accessed 2025-01-23).

[42] Hapka M., Pastorczak E., Pernal K. (2024). Self-Adapting Short-Range Correlation
Functional for Complete Active Space-Based Approximations. J. Phys. Chem. A.

[43] Modrzejewski, M. gammcor-integrals library. GitHub, 2022, https://github.com/modrzejewski/gammcor-integrals (accessed 2025-03-28).

[44] Sun Q., Zhang X., Banerjee S., Bao P., Barbry M., Blunt N. S., Bogdanov N. A., Booth G. H., Chen J., Cui Z.-H. (2020). Recent developments
in the PySCF program package. J. Chem. Phys..

[45] Dunning T. H. (1989). Gaussian basis
sets for use in correlated molecular
calculations. I. The atoms boron through neon and hydrogen. J. Chem. Phys..

[46] Davidson E. R., Hagstrom S. A., Chakravorty S. J., Umar V. M., Fischer C. F. (1991). Ground-state
correlation energies for two- to ten-electron atomic ions. Phys. Rev. A.

[47] Halkier A., Helgaker T., Jørgensen P., Klopper W., Koch H., Olsen J., Wilson A. K. (1998). Basis-set
convergence in correlated
calculations on Ne, N_2_, and H_2_O. Chem. Phys. Lett..

[48] Peterson K. A., Adler T. B., Werner H.-J. (2008). Systematically convergent
basis sets
for explicitly correlated wavefunctions: The atoms H, He, B-Ne, and
Al-Ar. J. Chem. Phys..

