# Supplementary material for: Correcting Basis Set Incompleteness in Wave Function Correlation Energy by Dressing Electronic Hamiltonian with an Effective Short-Range Interaction
Source: J Phys Chem Lett. 2025 Jun 17;16(25):6489–99. doi: 10.1021/acs.jpclett.5c01070 (PMC12207671; doi:10.1021/acs.jpclett.5c01070)
Supplement: Supplementary file 1 [file jz5c01070_si_001.pdf]

Supporting Information for: Correcting basis set incompleteness in wave function correlation energy by dressing electronic Hamiltonian with an effective short-range interaction

Michał Hapka,† Aleksandra Tucholska,‡ Marcin Modrzejewski,† Pavlo Golub,¶ Libor Veis,¶ and Katarzyna Pernal*,‡

†*University of Warsaw, Faculty of Chemistry, ul. L. Pasteura 1, 02-093 Warsaw, Poland*

‡*Institute of Physics, Lodz University of Technology, ul. Wolczanska 217/221, 93-005 Lodz, Poland*

¶*J. Heyrovský Institute of Physical Chemistry, Academy of Sciences of the Czech Republic, v.v.i., Dolejškova 3, 18223 Prague 8, Czech Republic*

E-mail: pernalk@gmail.com

Section 1 DBBSC from the perturbation theory

Recall the DBBSC has been equalized with the multideterminantal short-range correlation functional corresponding to a basis set-specific long-range interaction $v_{ee}^{\mathrm{LR},\mathcal{B}}$

$$\varepsilon_{\mathcal{B}}^{\mathrm{DBBSC}} = E_{\mathrm{c,md}}^{\mathrm{sr}}[\rho^{\mathcal{B}}] = \left\langle \Psi|\hat{T} + \hat{V}_{ee}|\Psi \right\rangle - \left\langle \Psi^{\mathrm{LR}}|\hat{T} + \hat{V}_{ee}|\Psi^{\mathrm{LR}} \right\rangle \quad , \tag{S.1}$$

(Ψ denotes an exact ground state in CBS of the exact Hamiltonian with the Coulomb interaction $\hat{V}_{ee}$, yielding the exact electron density ρ_0) under the condition that a given density obtained in a basis set $\mathcal{B}$ is equal to the exact density (in the complete basis set limit) ρ_0

$$\rho^{\mathcal{B}} = \rho_0 \tag{S.2}$$

The wavefunction Ψ^{LR} is en eigenfunction of an interacting Hamiltonian where Coulomb electron interaction is replaced with $v_{ee}^{\text{LR},\mathcal{B}}$. The cuspless wavefunction Ψ^{LR} is rapidly convergent with the basis set size and it is assumed that it is converged in a given basis set $\mathcal{B}$, which allows us to write

$$\varepsilon_{\mathcal{B}}^{\text{DBBSC}} = \left\langle \Psi | \hat{H} | \Psi \right\rangle - \left\langle \Psi_{\mathcal{B}}^{\text{LR}} | \hat{H} | \Psi_{\mathcal{B}}^{\text{LR}} \right\rangle \tag{S.3}$$

where it has been used that $\Psi_{\mathcal{B}}^{\text{LR}}$ is a ground state of the Hamiltonian $\hat{H}_{\mathcal{B}}^{\text{LR}}$

$$\hat{H}_{\mathcal{B}}^{\text{LR}} \Psi_{\mathcal{B}}^{\text{LR}} = E_{\mathcal{B}}^{\text{LR}} \Psi_{\mathcal{B}}^{\text{LR}} \tag{S.4}$$

$$\hat{H}_{\mathcal{B}}^{\text{LR}} = \sum_{pq}^{\mathcal{B}} \tilde{h}_{pq} \; \hat{a}_p^\dagger \hat{a}_q + \frac{1}{2} \sum_{pqrs}^{\mathcal{B}} g_{pqrs}^{\text{LR},\mathcal{B}} \; \hat{a}_p^\dagger \hat{a}_q^\dagger \hat{a}_s \hat{a}_r \tag{S.5}$$

$$g_{pqrs}^{\text{LR},\mathcal{B}} = \langle pq | v_{ee}^{\text{LR},\mathcal{B}} | rs \rangle \tag{S.6}$$

including a one-electron part $\tilde{h}$ that by definition fixes electron density to be equal to the exact one

$$\rho_{\Psi_{\mathcal{B}}^{\text{LR}}} = \rho_0 \tag{S.7}$$

Consider the exact Hamiltonian in the complete basis set,

$$\hat{H} = \sum_{pq}^{\text{CBS}} h_{pq} \; \hat{a}_p^\dagger \hat{a}_q + \frac{1}{2} \sum_{pqrs}^{\text{CBS}} g_{pqrs} \; \hat{a}_p^\dagger \hat{a}_q^\dagger \hat{a}_s \hat{a}_r \quad . \tag{S.8}$$

(g_{pqrs} denote two-electron integrals with Coulomb interaction) and denote a difference of $\hat{H}$ and $\hat{H}_{\mathcal{B}}^{\mathrm{LR}}$ by $\hat{H}'$

$$\hat{H}' = \hat{H} - \hat{H}_{\mathcal{B}}^{\mathrm{LR}} \quad . \tag{S.9}$$

Taking $\hat{H}_{\mathcal{B}}^{\mathrm{LR}}$ as a zeroth-order Hamiltonian, and $\Psi_{\mathcal{B}}^{\mathrm{LR}}$ as a zeroth-order state:

$$\hat{H}^{(0)} = \hat{H}_{\mathcal{B}}^{\mathrm{LR}} \tag{S.10}$$

$$\Psi^{(0)} = \Psi_{\mathcal{B}}^{\mathrm{LR}} \tag{S.11}$$

$$E^{(0)} = E_{\mathcal{B}}^{\mathrm{LR}} = \left\langle \Psi_{\mathcal{B}}^{\mathrm{LR}} | \hat{H}^{(0)} | \Psi_{\mathcal{B}}^{\mathrm{LR}} \right\rangle \quad . \tag{S.12}$$

and applying the perturbation theory to a Hamiltonian $\hat{H}(\alpha) = \hat{H}_{\mathcal{B}}^{\mathrm{LR}} + \alpha \hat{H}'$, yields the exact energy in orders of α

$$\begin{aligned} E_{exact} &= E^{(0)} + E^{(1)} + E^{(2)} + ... \\ &= \left\langle \Psi_{\mathcal{B}}^{\mathrm{LR}} | \hat{H} | \Psi_{\mathcal{B}}^{\mathrm{LR}} \right\rangle + E^{(2)} + ... \end{aligned} \tag{S.13}$$

A comparison with Eq.(S.3) yields

$$\varepsilon_{\mathcal{B}}^{\mathrm{DBBSC}} = E^{(2)} + ... \tag{S.14}$$

and it is evident that $\varepsilon_{\mathcal{B}}^{\mathrm{DBBSC}}$ follows in orders, beginning with the second-order with respect to $\hat{H}'$. After noticing that (i) $\hat{H}'$ acts on states of the Hamiltonian $\hat{H}_{\mathcal{B}}^{\mathrm{LR}}$, (ii) the short-range interaction operator is complementary to the long-range interaction namely,

$$g_{pqrs} = g_{pqrs}^{\mathrm{LR},\mathcal{B}} + g_{pqrs}^{\mathrm{SR},\mathcal{B}} \tag{S.15}$$

(iii) one-electron operators in $\hat{H}'$ (difference of h and $\tilde{h}$) are local operators and the perturbing operator does not affect electron density, see Eq.(S.7), $\hat{H}'$ can be written as

$$\hat{H}' = \frac{1}{2} \sum_{pqrs}^{\mathcal{B}} g_{pqrs}^{\mathrm{SR},\mathcal{B}} \; \hat{a}_p^\dagger \hat{a}_q^\dagger \hat{a}_s \hat{a}_r \tag{S.16}$$

We conclude therefore that the DBBSC correction could be obtained from the perturbation theory using the perturbing operator $\hat{H}'$ given above. This operator is of the same form as the one used in the CBS[H] method. In DBBSC an approximate functional is used to sum correlation to infinite order. In CBS[H] used together with AC0 and NEVPT2 methods the basis set correction is accounted for at the second order.

Section 2 Results in cc-pVXZ basis sets

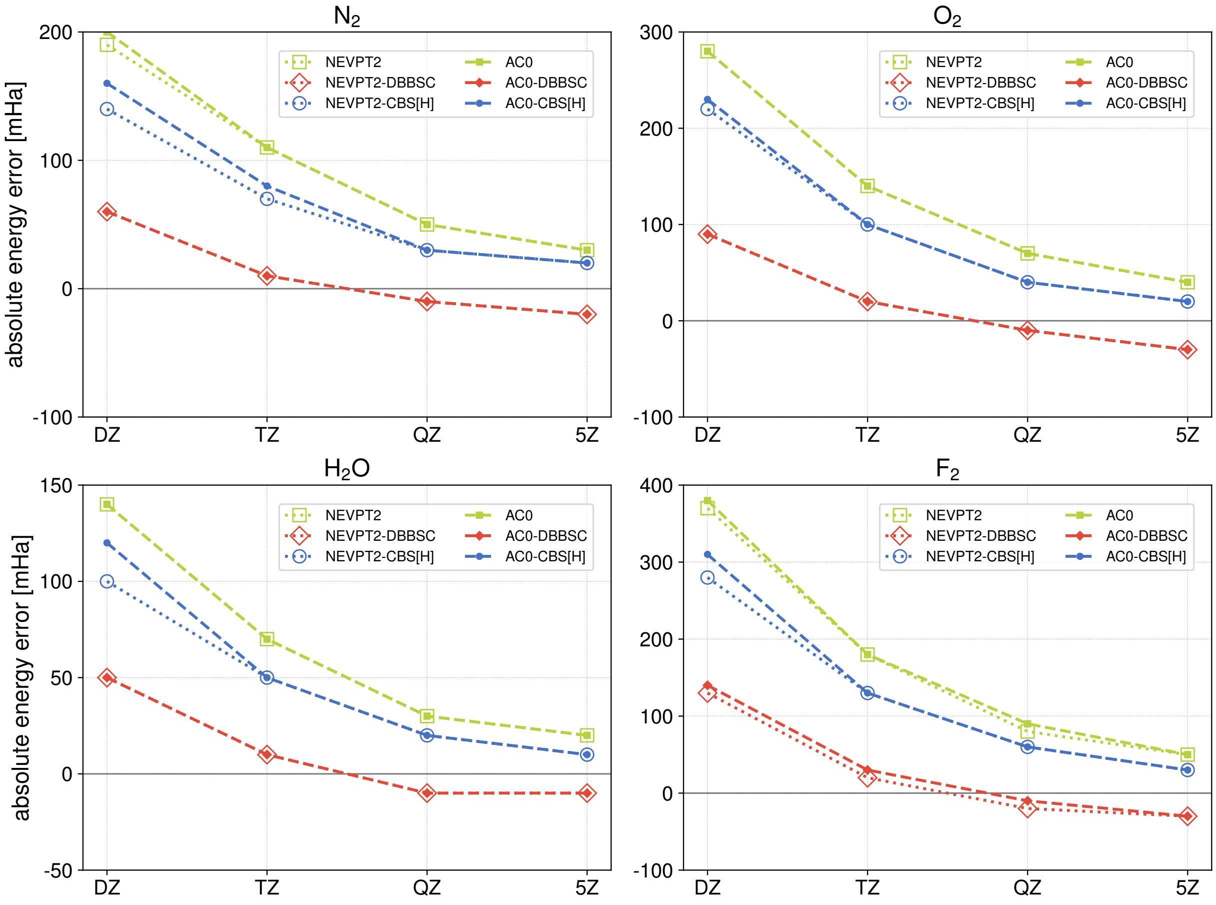

Figure S1: Absolute energy errors as a function of the cardinal number X for molecules in dissociation geometries.

Table S1: Absolute energy errors for molecules in equilibrium geometries as a function of the cardinal number X. Energy unit is mHa.

System	basis	NEVPT2	NEVPT2-DBBSC	NEVPT2-CBS[H]	AC0	AC0C-DBBSC	AC0-CBS[H]
N_2	DZ	233.27	64.91	182.20	238.42	70.06	191.47
	TZ	122.15	12.34	83.67	123.73	13.93	84.41
	QZ	60.41	-12.76	34.96	60.73	-12.43	35.65
	5Z	37.02	-21.37	22.03	37.02	-21.37	21.29
H_2O	DZ	174.64	60.76	129.55	180.66	66.84	142.68
	TZ	81.46	11.14	51.65	83.47	13.17	55.00
	QZ	37.90	-6.71	21.89	38.70	-5.89	22.06
	5Z	21.14	-12.95	11.63	21.54	-12.54	11.83
O_2	DZ	304.42	102.48	232.48	306.51	104.57	244.44
	TZ	149.51	17.92	99.96	150.66	19.08	103.44
	QZ	71.00	-14.38	40.89	71.53	-13.86	42.49
	5Z	41.09	-25.79	22.78	41.34	-25.53	23.69
F_2	DZ	381.00	132.82	282.78	398.30	150.11	320.71
	TZ	177.89	19.34	124.58	186.58	28.04	133.99
	QZ	81.33	-19.63	52.32	87.13	-13.83	56.49
	5Z	44.71	-32.62	27.99	49.64	-27.69	31.56
CH_2	DZ	95.66	24.79	77.36	98.16	27.29	77.87
singlet	TZ	49.82	4.30	34.10	50.62	5.10	32.96
	QZ	25.65	-5.63	15.49	25.96	-5.32	14.57
	5Z	16.30	-9.13	9.78	16.44	-8.99	9.12
CH_2	DZ	90.90	24.93	73.39	93.09	27.12	75.07
triplet	TZ	48.06	4.78	33.11	48.74	5.47	32.54
	QZ	25.14	-5.06	15.08	25.39	-4.80	15.00
	5Z	16.29	-8.52	10.01	16.42	-8.40	9.75
MUE	DZ	213.31	68.45	162.96	219.19	74.33	175.37
	TZ	104.81	11.64	71.18	107.30	14.13	73.73
	QZ	50.24	10.69	30.11	51.57	9.36	31.04
	5Z	29.42	18.40	17.37	30.40	17.42	17.87

CAS used: N_2– CAS(8,10); H_2O – CAS(8,6); O_2–(8,6); F_2– (14,8); CH_2– (6,6).
Geometry: N_2– R_{eq} = 2.07 a.u.; H_2O – R_{eq} = 1.809 a.u.; O_2–R_{eq} = 2.282 a.u.; F_2– R_{eq} = 2.730 a.u; CH_2– R_{eq} = 2.09 a.u, $\theta = 102.3°/133°$ singlet/triplet.

Table S2: Absolute energy errors for molecules in dissociation (linearized in case of CH_2) geometries as a function of the cardinal number X. Energy unit is mHa.

System	basis	NEVPT2	NEVPT2-DBBSC	NEVPT2-CBS[H]	AC0	AC0C-DBBSC	AC0-CBS[H]
N_2	DZ	194.06	57.04	144.60	198.37	61.34	158.68
	TZ	108.64	11.13	72.89	109.91	12.40	77.89
	QZ	53.11	-12.43	30.97	53.46	-12.09	32.46
	5Z	33.72	-20.75	19.86	33.83	-20.63	20.24
H_2O	DZ	140.38	45.91	103.56	144.81	50.35	118.51
	TZ	71.02	7.86	47.72	72.45	9.30	50.63
	QZ	33.62	-7.49	20.18	34.11	-7.00	20.57
	5Z	19.71	-12.93	11.63	19.89	-12.75	11.45
O_2	DZ	281.81	91.83	217.02	278.75	88.77	228.82
	TZ	142.80	16.08	96.69	142.79	16.07	101.75
	QZ	67.65	-14.77	39.94	67.83	-14.59	42.08
	5Z	39.74	-25.66	22.93	39.85	-25.55	23.83
F_2	DZ	371.11	128.75	276.35	383.79	141.43	312.87
	TZ	179.06	22.99	127.94	182.77	26.70	133.65
	QZ	84.41	-15.11	56.67	85.14	-14.39	55.95
	5Z	48.68	-27.84	32.61	48.41	-28.11	30.86
CH_2	DZ	104.10	31.09	79.63	106.82	33.80	84.21
singlet	TZ	53.08	6.58	32.55	54.02	7.53	33.89
	QZ	27.78	-4.24	14.27	28.17	-3.85	15.00
	5Z	17.90	-8.14	9.26	18.08	-7.96	9.48
CH_2	DZ	92.93	27.05	73.50	95.05	29.18	76.83
triplet	TZ	48.76	5.47	32.13	49.43	6.15	32.86
	QZ	25.85	-4.46	15.09	26.10	-4.20	15.43
	5Z	17.00	-8.01	10.15	17.12	-7.89	10.26
MUE	DZ	197.40	63.61	149.11	201.26	67.48	163.32
	TZ	100.56	11.69	68.32	101.90	13.02	71.78
	QZ	48.74	9.75	29.52	49.13	9.35	30.25
	5Z	29.46	17.22	17.74	29.53	17.15	17.69

CAS used: N_2– CAS(8,10); H_2O – CAS(8,6); O_2–(8,6); F_2– (14,8); CH_2– (6,6). Geometry : N_2– $R = 10.000$ a.u.; H_2O – $R = 9.500$ a.u.; O_2–$R = 10.000$ a.u.; F_2– $R = 9.500$ a.u; CH_2– $R = 2.09$ a.u, $\theta = 180°$.

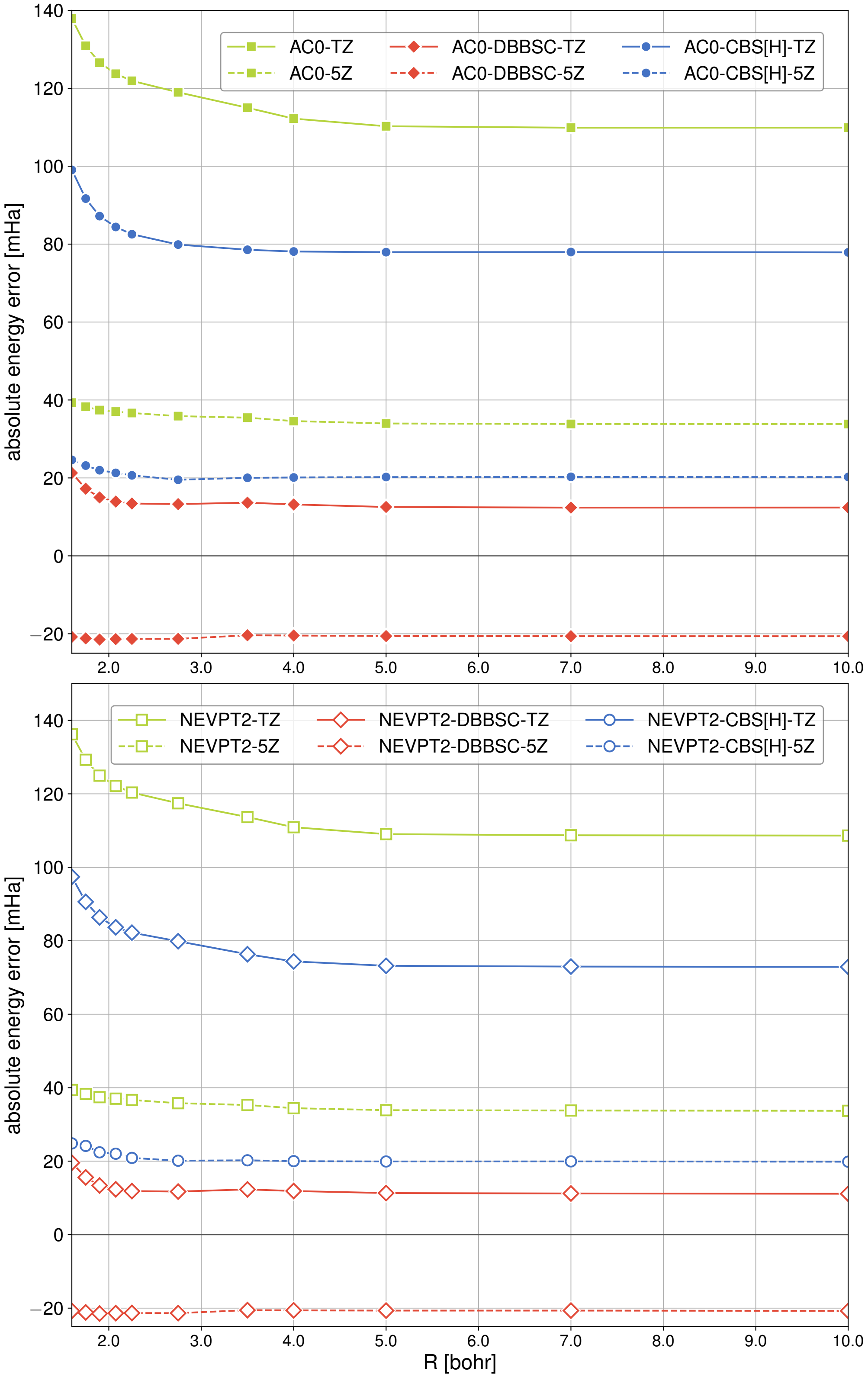

Figure S2: Errors in absolute energies for N_2

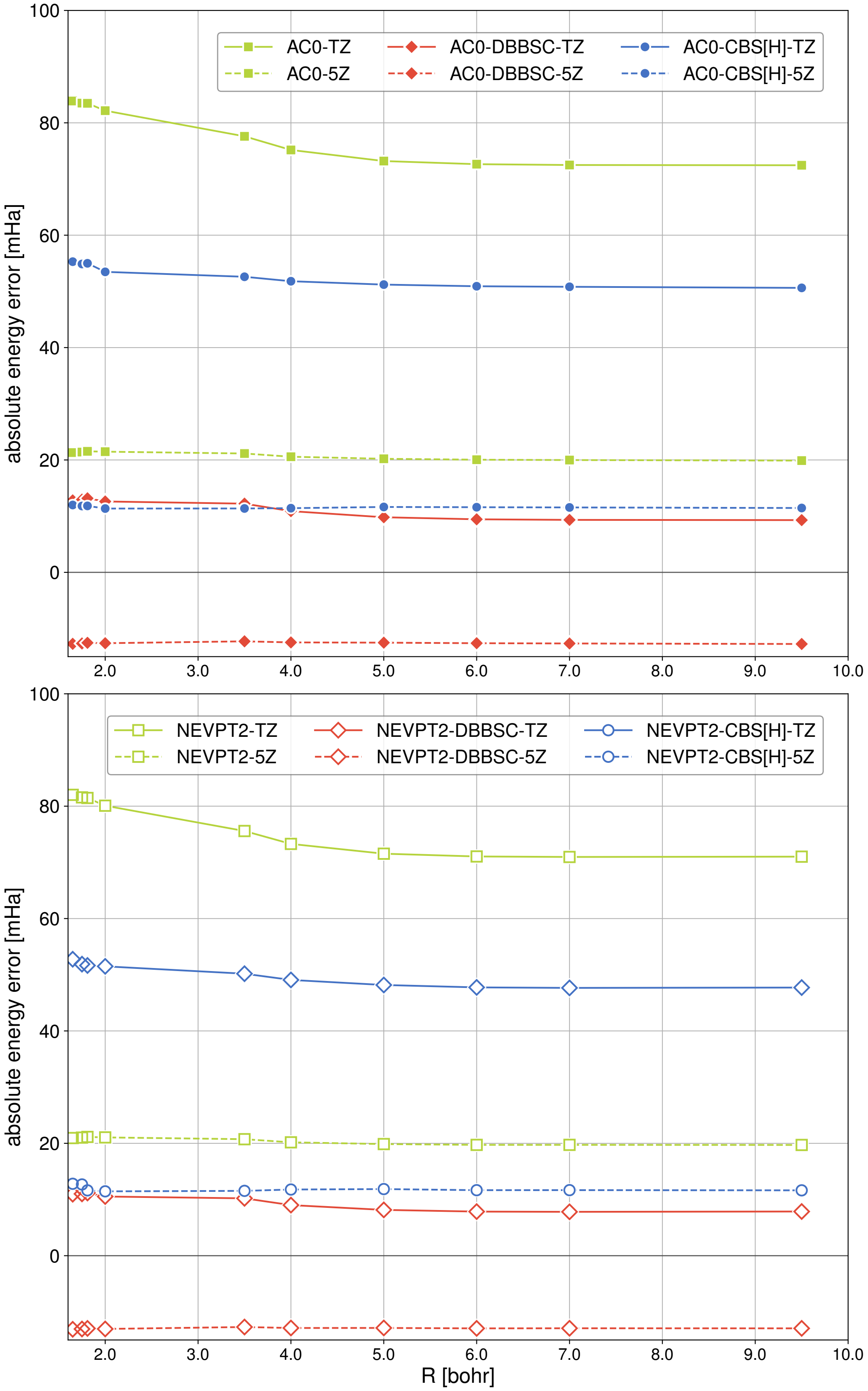

Figure S3: Errors in absolute energies for H_2O

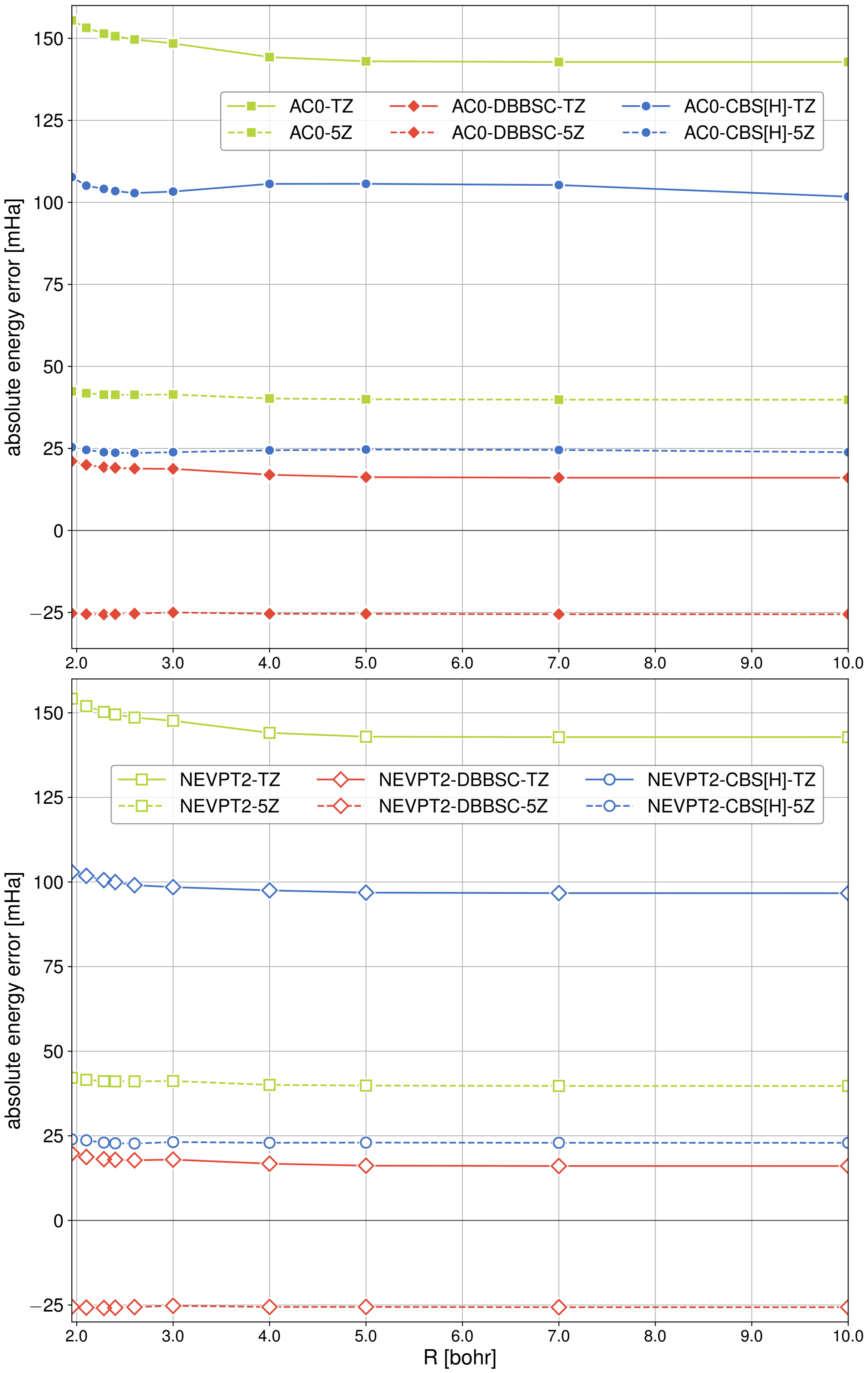

Figure S4: Errors in absolute energies for O_2

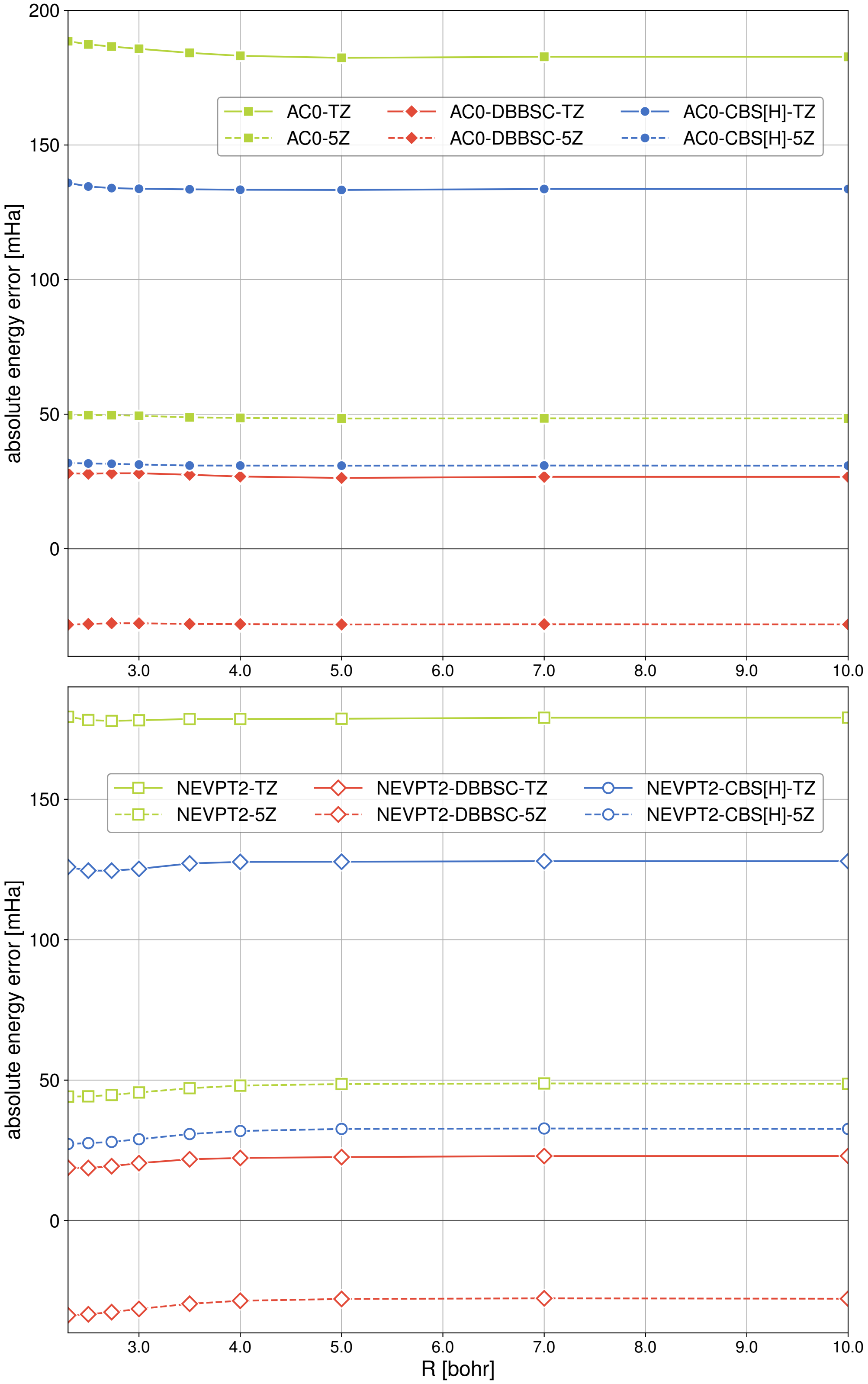

Figure S5: Errors in absolute energies for F_2

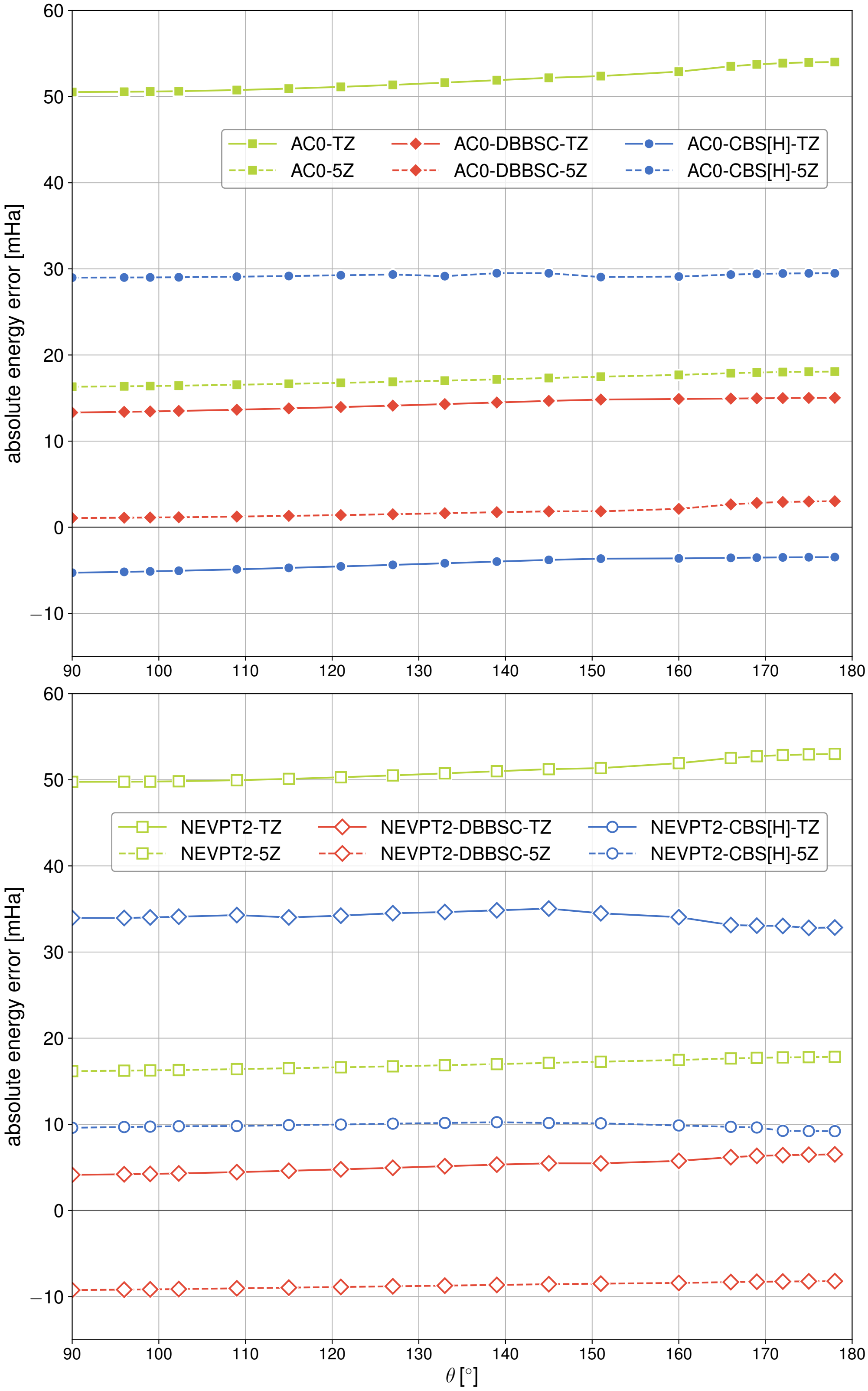

Figure S6: Errors in absolute energies for CH_2 in S state.

Section 3 Results in cc-pVXZ-F12 basis sets

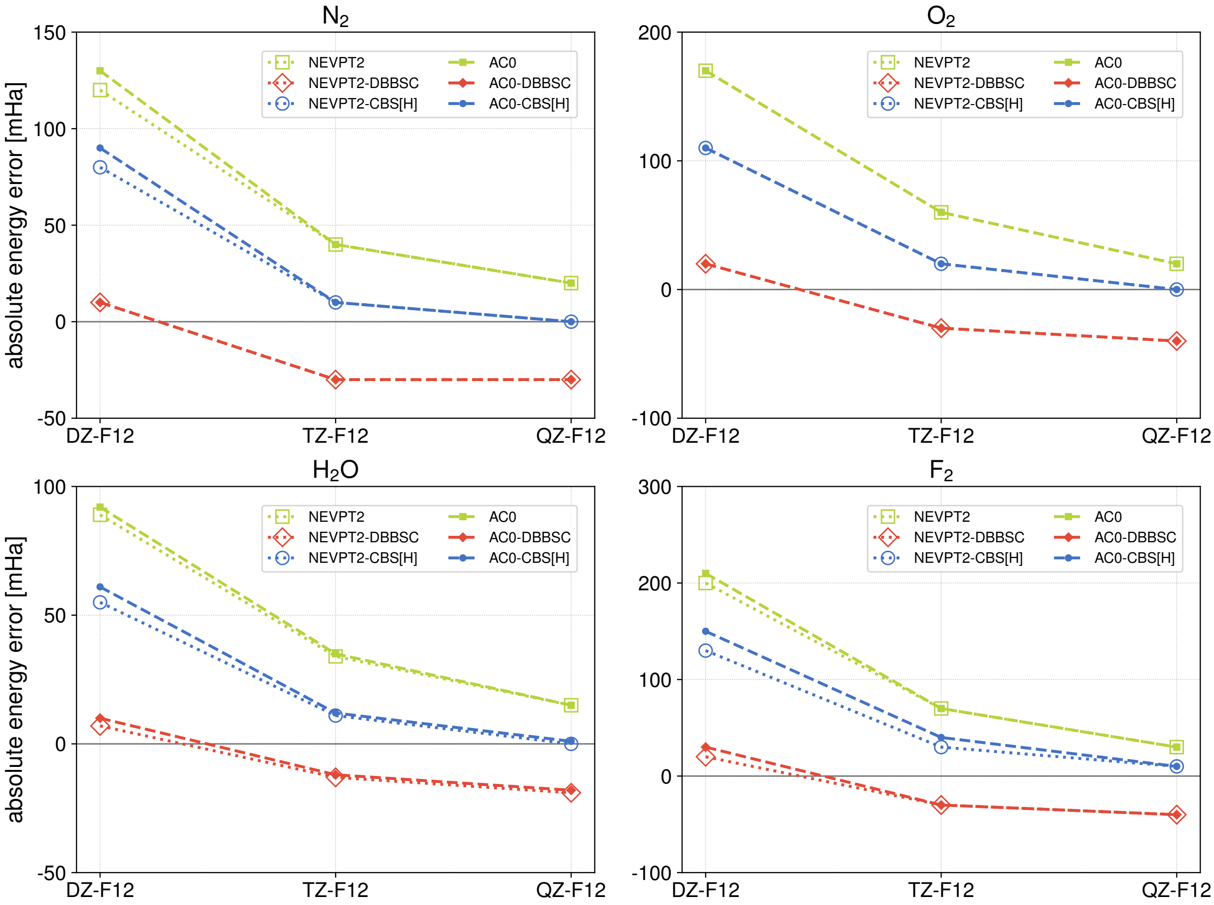

Figure S7: Absolute energy errors for molecules in equilibrium geometries as a function of the cardinal number X.

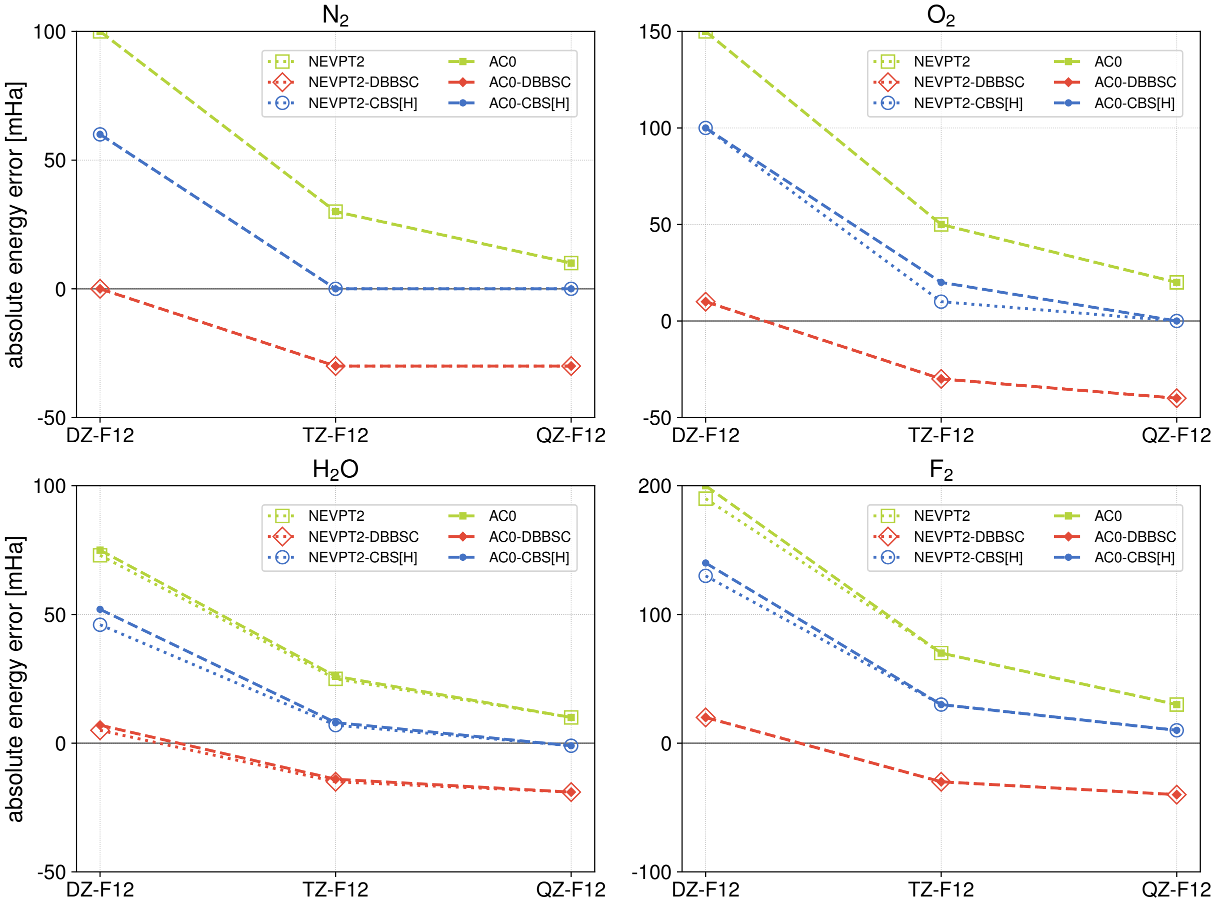

Figure S8: Absolute energy errors as a function of the cardinal number X for molecules in dissociation geometries.

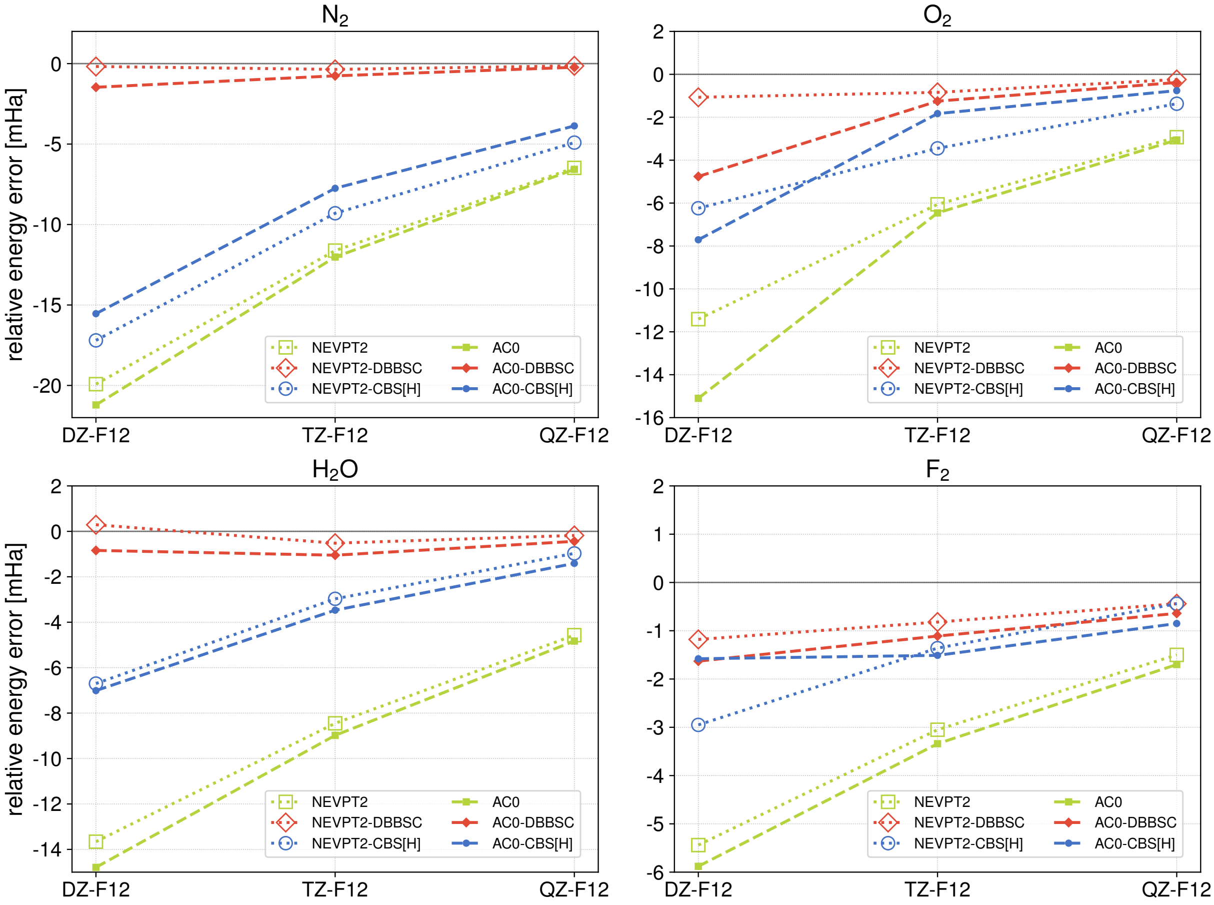

Figure S9: Errors of dissociation energies as a function of the cardinal number X.

Table S3: Dissociation energy errors as a function of the cardinal number X. Energy unit is mHa.

System	basis	NEVPT2	NEVPT2-DBBSC	NEVPT2-CBS[H]	AC0	AC0C-DBBSC	AC0-CBS[H]
N_2	DZ-F12	-19.92	-0.18	-17.20	-21.21	-1.47	-15.54
	TZ-F12	-11.62	-0.36	-9.30	-12.02	-0.76	-7.75
	QZ-F12	-6.48	-0.14	-4.91	-6.57	-0.23	-3.87
H_2O	DZ-F12	-13.66	0.29	-6.70	-14.79	-0.84	-7.01
	TZ-F12	-8.45	-0.52	-2.97	-8.98	-1.05	-3.47
	QZ-F12	-4.57	-0.18	-0.97	-4.83	-0.44	-1.41
O_2	DZ-F12	-11.41	-1.07	-6.24	-15.10	-4.76	-7.71
	TZ-F12	-6.06	-0.84	-3.45	-6.46	-1.25	-1.83
	QZ-F12	-2.93	-0.24	-1.37	-3.06	-0.38	-0.76
F_2	DZ-F12	-5.44	-1.18	-2.95	-5.88	-1.63	-1.58
	TZ-F12	-3.05	-0.82	-1.36	-3.34	-1.11	-1.51
	QZ-F12	-1.50	-0.44	-0.44	-1.70	-0.64	-0.85

CAS used: N_2– CAS(8,10); H_2O – CAS(8,6); O_2–(8,6); F_2– (14,8). R_{eq} : N_2– 2.07 a.u.; H_2O – 1.809 a.u.; O_2–2.282 a.u.; F_2– 2.730 a.u. R_x : N_2– 10.000 a.u.; H_2O – 9.500 a.u.; O_2–10.000 a.u.; F_2– 9.500 a.u.

Table S4: Absolute energy errors for molecules in equilibrium geometries as a function of the cardinal number X. Energy unit is mHa.

System	basis	NEVPT2	NEVPT2-DBBSC	NEVPT2-CBS[H]	AC0	AC0C-DBBSC	AC0-CBS[H]
N_2	DZ-F12	121.72	5.71	80.80	125.06	9.05	85.21
	TZ-F12	41.84	-26.25	12.54	42.81	-25.27	13.03
	QZ-F12	19.42	-31.30	0.41	19.79	-30.93	0.11
H_2O	DZ-F12	89.23	6.99	55.29	92.34	10.10	60.73
	TZ-F12	34.28	-13.48	10.69	35.37	-12.39	12.25
	QZ-F12	14.62	-18.64	0.26	15.07	-18.19	0.58
O_2	DZ-F12	165.15	17.54	108.06	166.70	19.09	114.41
	TZ-F12	57.60	-27.65	18.42	58.24	-27.01	20.45
	QZ-F12	23.33	-37.29	-0.69	23.59	-37.03	0.30
F_2	DZ-F12	201.09	19.02	133.63	207.44	25.37	146.35
	TZ-F12	72.31	-28.83	34.36	74.47	-26.67	35.64
	QZ-F12	32.25	-40.99	8.81	33.20	-40.04	8.57

CAS used: N_2– CAS(8,10); H_2O – CAS(8,6); O_2–(8,6); F_2– (14,8). R_{eq} : N_2– 2.07 a.u.; H_2O – 1.809 a.u.; O_2–2.282 a.u.; F_2– 2.730 a.u.

Table S5: Absolute energy errors for molecules in dissociation geometries as a function of the cardinal number X. Energy unit is mHa.

System	basis	NEVPT2	NEVPT2-DBBSC	NEVPT2-CBS[H]	AC0	AC0C-DBBSC	AC0-CBS[H]
N_2	DZ-F12	96.59	0.31	58.39	98.64	2.36	64.46
	TZ-F12	29.79	-27.04	2.81	30.37	-26.46	4.85
	QZ-F12	12.89	-31.49	-4.55	13.17	-31.21	-3.80
H_2O	DZ-F12	73.41	5.12	46.42	75.38	7.10	51.55
	TZ-F12	25.13	-14.69	7.02	25.69	-14.14	8.08
	QZ-F12	9.98	-18.88	-0.77	10.18	-18.69	-0.90
O_2	DZ-F12	148.73	11.45	96.81	146.59	9.32	101.69
	TZ-F12	51.06	-28.98	14.48	51.29	-28.75	18.12
	QZ-F12	20.40	-37.54	-2.07	20.53	-37.41	-0.46
F_2	DZ-F12	193.19	15.38	128.22	199.10	21.29	142.31
	TZ-F12	69.04	-29.88	32.78	70.91	-28.01	33.90
	QZ-F12	30.75	-41.43	8.36	31.50	-40.68	7.72

CAS used: N_2– CAS(8,10); H_2O – CAS(8,6); O_2–(8,6); F_2– (14,8).
R_x : N_2– 10.000 a.u.; H_2O – 9.500 a.u.; O_2–10.000 a.u.; F_2– 9.500 a.u.
